# miR-195b is required for proper cellular homeostasis in the elderly

**DOI:** 10.1038/s41598-024-51256-8

**Published:** 2024-01-08

**Authors:** Maria del Mar Muñoz-Gallardo, Carlos Garcia-Padilla, Cristina Vicente-Garcia, Jaime Carvajal, Amelia Arenega, Diego Franco

**Affiliations:** 1 Cardiovascular Development Group, Department of Experimental Biology, University of Jaen, Jaen, Spain; 2 Department of Anatomy, Embryology and Zoology, School of Medicine, University of Extremadura, Badajoz, Spain; 3 Andalusian Centre of Developmental Biology (CABD-CSIC-UPO-JA), Seville, Spain; 4 Fundación Medina, Granada, Spain

**Keywords:** Cell signalling, Mechanisms of disease, Post-translational modifications, Senescence

## Abstract

Over the last decade we have witnessed an increasing number of studies revealing the functional role of non-coding RNAs in a multitude of biological processes, including cellular homeostasis, proliferation and differentiation. Impaired expression of non-coding RNAs can cause distinct pathological conditions, including herein those affecting the gastrointestinal and cardiorespiratory systems, respectively. miR-15/miR-16/miR-195 family members have been broadly implicated in multiple biological processes, including regulation of cell proliferation, apoptosis and metabolism within distinct tissues, such as heart, liver and lungs. While the functional contribution of miR-195a has been reported in multiple biological contexts, the role of miR-195b remains unexplored. In this study we dissected the functional role of miR-195b by generating CRISPR-Cas9 gene edited miR-195b deficient mice. Our results demonstrate that miR-195b is dispensable for embryonic development. miR-195b^−/−^ mice are fertile and displayed no gross anatomical and/or morphological defects. Mechanistically, cell cycle regulation, metabolism and oxidative stress markers are distinctly impaired in the heart, liver and lungs of aged mice, a condition that is not overtly observed at midlife. The lack of overt functional disarray during embryonic development and early adulthood might be due to temporal and tissue-specific compensatory mechanisms driven by selective upregulation miR-15/miR-16/miR-195 family members. Overall, our data demonstrated that miR-195b is dispensable for embryonic development and adulthood but is required for cellular homeostasis in the elderly.

## Introduction

Non-coding RNAs are broadly classified according to their length into two distinct categories. Small non-coding RNAs, including piwiRNA, snoRNA and microRNAs, are shorter than 200 nucleotides while long non-coding RNAs, including therein lincRNAs and circRNAs, are longer than 200 nucleotides^1^. Among the small non-coding RNAs, microRNAs represent the most abundantly expressed and functionally characterized subgroup. MicroRNAs are encoded in the nucleus, by transcription of precursors microRNAs molecules that are normally transcribed by RNA polymerase II^2^. In certain genomic localization, microRNAs are clustered in such a way that the primary transcript contains multiple microRNA precursors and this transcript is dubbed pri-miRNA. Pri-miRNAs are then processed by RNAses such as Drosha and Dgcr8 to generate distinct pre-miRNA molecules, that are subsequently exported to the cytoplasm by the exportin-5/Ran protein complex^2^. Within the cytoplasm the pre-miRNA is processed into a mature microRNA duplex by Dicer RNAse and loaded into the RISC complex in which one strand is degraded. The mature single-stranded microRNA molecule within the RISC complex is able now to scan other RNA molecules for sequence homology of its seed sequence provoking RNA target cleavage, translation repression and/or RNA deadenylation^2^. Importantly, microRNAs are highly conserved during evolution, ranging from *C. elegans* to humans^3^.

Over the last decade we have witnessed an increasing number of studies revealing the functional role of non-coding RNAs in a multitude of biological processes, including cellular homeostasis, proliferation and differentiation among others^4–6^. Impaired expression of non-coding RNAs can cause distinct pathological conditions, including herein those affecting to the gastrointestinal tract^7,8^ and the cardiorespiratory system^9,10^ among others, while pathological conditions are also associated with non-coding RNA dysregulation^11^.

Functional contribution of microRNAs to embryonic development and homeostasis have been extensively reported using gene targeting approaches^12^. Several microRNAs such as miR-1-1, miR-133 and miR-126 are essential for cardiovascular development^13,14^, respectively, while miR-9 regulates neurogenesis^15^. Similarly, distinct microRNAs have been reported to play essential roles in adulthood, such as miR-7 regulating β-pancreatic cell function^16^, miR-22 modulating cardiac contractile function^17^ and miR-184 regulating epidermal differentiation^18^. Importantly, several microRNAs share the same seed sequence and thus can potentially regulate the same transcript targets, such as miR-15/miR16/miR-195 microRNA family. miR-15, miR-16 and miR-195 have been broadly implicated in multiple biological processes, such as regulating cell proliferation^19,20^, apoptosis^21,22^ and metabolism^23^, within distinct tissues, such as the heart^24,25^, liver^26,27^ and lungs^28–30^. Importantly, two distinct miR-195 isoforms are found in the mouse genome; (a) miR-195a located in chromosome 11 adjacent to miR-497a and miR-497b, and (b) miR-195b on chromosome 2 and located in a gene desert. While the functional contribution of miR-195a has been reported in multiple biological contexts^19–30^, particularly in gain-of-function models, the role of miR-195b remains unexplored. In this study we aim to dissect the functional role of miR-195b by generating CRISPR-Cas9 gene edited miR-195b deficient mice. Our results demonstrate that genetic deletion of miR-195b is dispensable for embryonic development. miR-195b knockout mice are fertile and displayed no gross anatomical and/or morphological defects during adulthood. Molecular analyses demonstrated nonetheless, that cell cycle regulation, metabolism and oxidative stress is distinctly impaired in the heart, liver and lungs of aged mice, a condition that is not overtly observed at midlife. Mechanistically, the lack of overt functional disarray during embryonic development and early adulthood might be due to temporal and tissue-specific compensatory mechanisms driven by selective upregulation of miR-195a, miR-15 and miR-16 expression. Overall, our data demonstrated that miR-195b is dispensable for embryonic development and adulthood but is required for cellular homeostasis in the elderly, particularly in the liver.

## Materials and methods

### Ethics statement

Experiments using animals were performed under protocols approved by the Universidad Pablo de Olavide Ethical Committee (Sevilla, Spain; protocol 22/09/2015/329) and by the Universidad de Jaén Ethical Committee (Jaé, Spain; protocol 18/11/14/155) in accordance with Spanish Royal Decree 53/2013, European Directive 2010/63/EU, and other relevant guidelines. This study is reported in accordance with ARRIVE guidelines (https://arriveguidelines.org).

### Animals (gene expression analyses)

CD1 mice were bred and embryos were collected at distinct embryonic day (E) ranging from E10.5 to E16.5. Pregnant females were euthanized by cervical dislocation. Subsequently, embryos were isolated, dissected and stored in liquid nitrogen until used. Similarly, adult CD1 mice (6 months) were euthanized by cervical dislocation and distinct tissues were isolated, dissected and stored in liquid nitrogen until used.

### Gene-editing, miR-195b deficient mice breeding

miR-195b deficient mice were obtained by CRISPR-Cas9 gene editing. Two CRISPR guide RNAs (sgRNAs) were designed using CRISPRSCAN^31^ at either side of miR-195b to provide a deletion encompassing the whole gene (Supplementary Fig. 1A). They were synthesized by in vitro transcription from DNA templates generated by fill-in PCR, as reported elsewhere^31^. Briefly, for each sgRNA, two complementary oligos were used. One of them was sgRNA-specific and contained the sequence for the T7 promoter, the CRISPR target sequence and 15 additional nucleotides complementary to the second oligo, which was universal and contained the constant sgRNA scaffold sequence. Each oligo pair, one per sgRNA to be generated, were subjected to PCR amplification to generate 117-bp PCR products using the following protocol: 3 min at 95 °C, 30 cycles of 30 s at 95 °C, 30 s at 58 °C and 20 s at 72 °C, and a final step of 5 min at 72 °C. Next, PCRs products were purified using the High Pure PCR Product Purification kit from Roche and then used as templates for in vitro transcription using the T7 Transcription kit from Roche, following the manufacturer’s instructions. The newly synthesized sgRNAs were treated with DNAseA to remove DNA contaminants, purified by ethanol precipitation and resuspended in DEPC H_2_O. Quantification was performed using Nanodrop and confirmed running serial dilutions of the samples in parallel with serial dilutions of brewer’s yeast tRNA (Roche) of known concentrations. Finally, sgRNA activity was evaluated using in vitro digestion assays IDAs^32^. Briefly, DNA templates containing the CRISPR-Cas9 target sites at either side or miR-195b were PCR-amplified. Then, PCR products were purified using the High Pure PCR Product Purification kit from Roche, and incubated for 3 h at 37 °C with Cas9 protein (20 ng/μL, Addgene vector #47327) and the corresponding sgRNA (2.5 ng/μL) in digestion buffer (20 mM HEPES pH 7.5, 150 mM KCl, 0.5 mM DTT, 0.1 mM EDTA, 10 mM MgCl2). Digestion products were run in a 1% agarose gel stained with ethidium bromide to assess cleavage efficiency. The sequence of the oligos used for sgRNAs generation, and for IDAs and genotyping are the following: miR-195b_5′guide (taatacgactcactataGGTAGATAAAGTAGCTTCTTgttttagagctagaa), mir-195b_3′guide (taatacgactcactataGGAGAAAATGCTGTCTTGGAgttttagagctagaa), universal_guide (aaaagcaccgactcggtgccactttttcaagttgataacggactagccttattttaacttgctatttctagctctaaaac), mir-195b_genotF (tggttgacctgcctctaaca) and mir-195b_genotR (tgaggcatccttatttgggta). IDA templates were generated with the same primers used for genotyping.

Cas9 mRNA (100 ng/μL, SBI) and sgRNA (10 ng/μL) were co-injected into the cytoplasm of CBA/C57Bl6 fertilized eggs and transferred to pseudopregnant CD1 females using standard methods. After weaning, F0 pups carrying deletions encompassing the miR-195b gene were detected by performing PCR with the same primers used for IDA template generation. Genomic DNA was obtained from ear biopsies. Deletions were confirmed by sequencing after TA cloning into pCR2.1 (Invitrogen) of PCR products (data not shown). Next, mutant F0 carriers were crossed with wild-type CBA/C57Bl6 hybrids to generate distinct miR-195b deficient mouse lines and to minimize potential off-target effects. miR-195b heterozygous were likewise genotyped to identify the specific deletion allele transmitted. Further, they were intercrossed with CBA/C57Bl6 wild type, establishing three distinct lines. Deletions included in all cases the miR-195b premiRNA sequence with distinct flanking sequences; total deletion length ranging from 450 to 600 bps, respectively (Supplementary Fig. 1B, C). Mice were bred to adulthood and processed for anatomical and histological examination. No increased mortality was observed in any of the three distinct miR-195b null transgenic mice. Therefore, we selected only one single line for further characterization of miR-195b deficiency. Three distinct conditions of miR-195b deficient mice were subsequently obtained, control miR-195b^+/+^ with no disruption of the miR-195b locus, heterozygous miR-195b mice (miR-195b^+/−^) and homozygous miR-195b null mice (miR-195b^−/−^); i.e. the deletion was maintained in heterozygosity and homozygosity in a CBA/C57Bl6 hybrid background.

### miR-195b deficient mice tissue sample collection

Control miR-195b^+/^, heterozygous miR-195b mice (miR-195b^+/−^) and homozygous miR-195b null mice (miR-195b^+/+^) adult mice were obtained at distinct time points ranging from postnatal 30 days to 420 days. Adult mice were euthanized by cervical dislocation and gross anatomical examination was performed. Subsequently, tissue samples from distinct organs were collected and processed for molecular analyses by liquid nitrogen snap frozen, or for histological analyses by fixation in 4% PFA at room temperature followed by graded ethanol dehydration and paraffin embedment.

### In situ hybridization ISH (whole-mount and tissue sections)

Embryos were collected at distinct embryonic stages as previously described, randing from E10-5 to E16.5, fixed for 2 h at 4 °C in 4% PFA, dehydrated in graded ethanol steps and stored at − 20 °C. Embryos were processed for whole-mount LNA-ISH was processed as previously described^33^. miR-195 LNA-labeled microRNA probe (miRCURY LNA™ Detection probe 5′-DIG and 3′-DIG labeled, Exiqon) was used to performed whole-mount LNA ISH. For ISH on tissue sections the embryos, previously embedded in paraffin, were sectioned (10 µm) and mounted into 3-aminopropyltriethoxysilane(AAS)-coated glasses. The Mlc2a (Myl7) riboprobe was used as positive internal hybridization control, as previously reported^34^, following the ISH protocol described by Lopez-Sanchez et al.^35^.

### Cell cultures and transfections assays

Immortalized murine atrial myocardial HL-1^36^, immortalized embryonic endocardial MEVEC^37^, 3T3 fibroblasts (ATCC), Hep-G2 (ATCC) and epicardial EPIC^38^ cells were used for miR-195b gain and loss of function assays, respectively. All cell lines were cultured in DMEM medium supplemented with 10% fetal bovine serum, 100 U/mL penicillin, 100 μg/mL streptomycin and 200 nM of L-glutamine in 100 cm^2^ culture disks at 37 °C in a humidified atmosphere of 5% CO_2_, respectively, except atrial HL1 cells that were culture in Claycomb medium and supplemented with 2 nM norepinephrine. Cells were fed every 2–3 days. microRNA 195 mimic (pre-miRNA 195) and microRNA 195 inhibitor (anti-miRNA 195) (Thermo-Fisher) transfections were carried out with Lipofectamine 2000 (Invitrogen), following the manufacturer’s guidelines. Briefly, 50 nM of pre-miRNA and/or anti-miRNA were applied to the different cell cultures (6 × 10^4^ cells per well) in antibiotic-free medium for 24 h, respectively. Subsequently, cells were collected by centrifugation and stored at -80 °C until used.

### Mitotracker and ROS labeling

The Hep-2G cell line was used to evaluate in vitro mitochondrial function and ROS activity, respectively. The number of active mitochondria was measured using Mitotracker Green FM labelling (Thermofisher #M7514), while ROS levels were measured using CellROX™ Deep Red labelling (Thermofisher # C10422), following in both cases, the manufacturer’s guidelines, respectively. Hep-2G cells (6 × 10^4^ cells per well) were transfected with microRNA 195b inhibitor (Thermo Fisher) as previously described and analyzed using a Leica TCS SP5 II confocal scanning laser microscope. Images were subsequently quantified using ImageJ software.

### Histochemistry and Immunohistochemistry

Tissue samples were collected from 30, 60, 120, 240 and 420 day-old mice corresponding to distinct genotypes (miR-195b^+/+^, miR-195b^+/−^ and miR-195b^−/−^), rinsed in PBS for 10 min at room temperature, and fixed with 4% PFA overnight at 4 °C. After fixation, the samples were rinsed three times (10 min each) in PBS at room temperature and then dehydrated in graded ethanol steps. Subsequently, dehydrated samples were paraffin embedded and cut into 10 µm sections which, in turn, were permeabilized with 1% Triton X-100 in PBS for 30 min at room temperature. Histological sections were routinely stained with H&E and picrosirius red. For immunohistochemical analyses, histological sections were incubated in PBS containing 5% goat serum and 1% bovine serum albumin (Sigma) overnight at 4 °C to block nonspecific binding sites. As primary antibody, monoclonal rabbit recombinant Anti-Ki67 (ab16667, Abcam) was used, diluted (1:200) in PBS, and applied to each section overnight at 4 °C. Subsequently, the sections were rinsed three times (for 1 h each) in PBS to remove excess primary antibody and incubated overnight at 4 °C with Alexa-Fluor 546 anti-rabbit (1:100; Invitrogen) as secondary antibody. After incubation with the secondary antibody, the sections were rinsed as described above and incubated with DAPI (1:1,000; Sigma) for 7 min at room temperature and rinsed three times in PBS for 5 min each. Finally, the sections were mounted with *Hydromount* medium and stored in PBS in darkness at 4 °C until analyzed using a Leica TCS SP5 II confocal scanning laser microscope. Negative controls, lacking primary antibody incubation, resulted in all cases is no detectable signal. Images were subsequently quantified using ImageJ software.

### SA-β-galactosidase staining and confocal analyses

The 3T3 cell line (ATCC) was used to analyze senescence associated (SA)-β-galactosidase activity in vitro as previously described^39^. 3T3 cells were transfected with anti-miR-195b (Thermofisher) or with a plasmid containing expression constructs for β-galactosidase as positive control, in antibiotic free medium for 24 h with Lipofectamine 2000 (Invitrogen), following the manufacturer’s guidelines. Additionally, oxygen peroxide (H_2_O_2_) administration was also applied as internal positive control for induced senescence. Blue-stained senescent cells were observed by light microscopy and manually counted.

Evaluation of SA-β-galactosidase expression in vivo was carried out by immunohistochemistry. A primary antibody against β-galactosidase (ThermoFisher Catalog # 200-901-036) was used, diluted (1:500) in PBS, and applied to each tissue sections overnight at 4 °C. Subsequently, sections were rinsed three times, for 1 h each, in PBS to remove excess primary antibody and incubated 3 h at room temperature with Alexa-Fluor 546 anti-chicken (1:100; Invitrogen, Catalog # A-11040) as secondary antibody. After secondary antibody incubation, the sections were rinsed as described before and incubated with DAPI (1:1,000; Sigma) for 7 min at room temperature and rinsed three times in PBS for 5 min each. Finally, the sections were mounted with *Hydromount* medium and stored in PBS in darkness at 4 °C until analyzed using a Leica TCS SP5 II confocal scanning laser microscope. Negative controls, lacking primary antibody incubation, resulted in all cases is no detectable signal.

### 3′UTR cloning and luciferase assays

*Ccnd1*, *Ccnd2*, *Ccnd3*, *Stim1* and *Atf6* 3′UTR constructs were PCR-amplified and cloned into the pMIR-REPORT vector. Primer sequences are provided in Supplementary Table 1. 3T3 fibroblasts (ATCC) were co-transfected with 100 ng of *Ccnd1*, *Ccnd2*, *Ccnd3*, *Stim1* and *Atf6* pMIR-Report luciferase vector, respectively, and 300 ng of pcLux vector control for internal normalization. Luciferase activity was measured 18 h after transfection using Pierce Gaussia Luciferase Flash Assay Kit (Thermo Fisher Scientific, Rockford, IL, USA) and normalized to pcLux vector control, using Pierce Cypridina Luciferase Flash Assay Kit (Thermo Fisher Scientific, Rockford, IL, USA). In all cases, transfections were carried out in triplicate and internal controls were measured on each assay.

### RNA isolation and cDNA synthesis

Total RNA was isolated using Trizol (Roche) according to manufacturer’s guidelines and DNase treated using RNase-Free DNase (Roche) for 1 h at 30 °C. In all cases, at least three distinct pooled samples were used to perform the corresponding RT-qPCR experiments.

For mRNA analyses, first strand cDNA was synthesized at 50 °C for 1 h using 1 μg of RNA, oligo-dT primers and Superscript III Reverse Transcriptase (Invitrogen) according to manufacturer’s guidelines. Negative controls to assess genomic contamination were performed for each sample, without reverse transcriptase, which resulted in all cases in no detectable amplification product. For microRNA expression analyses, 20 ng of total RNA was used for retro-transcription with Universal cDNA Synthesis Kit II (Exiqon) and the resulting cDNA was diluted 1/80, following manufacture′s guidelines.

### RT-qPCR analyses (mRNA and microRNA)

SyBR Green-based RT-qPCR was performed in a Mx3005Tm QPCR System with an MxPro QPCR Software 3.00 (Stratagene). Reactions were performed in 96-well plates with optical sealing tape (Cultek) in 20 μL total volume containing SYBR Green Mix (Finnzymes) and the corresponding cDNA.

For mRNA analyses, two internal controls, mouse *Gusb* and *Gapdh* mRNAs, were used in parallel for each run and represented as previously described^40^. Amplification conditions were as follows: denaturalization step of 95 °C for 10 min, followed by 40 cycles of 95 °C for 30 s, 60 °C for 30 s, 72 °C for 30 s; with final elongation step of 72 °C for 10 min. All primers were designed to span exon-exon boundaries using online Primer3 software Primer3input (http://bioinfo.ut.ee/primer3-0.4.0/). Primer sequences are provided in Supplementary Table 1.

For microRNA expression analyses, 20 ng of total RNA was used for retrotranscription with Universal cDNA Synthesis Kit II (Exiqon) and the resulting cDNA was diluted 1/80. Real time PCR experiments were performed with 1 μL of diluted cDNA, ExiLENT SYBR Green master mix (Exiqon) and corresponding primer sets.

No amplifications were observed in PCR control reactions containing only water as the template. Each PCR reaction was performed at least three times to obtain representative averages. The Livak method^41^ was used to analyze the relative quantification RT-PCR data and normalized in all cases taking as 100% the wild-type (control) value, as previously described^42^.

### miR-195b Taqman RT-qPCR analyses

Absolute quantification of miR-195b was performed using miR-195b Taqman probes (A25576, ThermoFisher) and related to cel-miR-39_3p calibration curve obtained also with Taqman probes (4,427,975, ThermoFisher). 5 ng of total RNA was used for retrotranscription with Taqman MicroRNA Reverse Transcription Kit (Applied Byosystems). Real time PCR experiments were performed with 1 μL of diluted cDNA, Taqman Fast Advanced Master Mix (Applied Byosistems)and corresponding Taqman probes. U6 was assessed as an internal positive control. Four distinct dilution series were assayed (1 to 1:1000) and the Ct values of the corresponding dilution series were used to generate the cel-miR-39_3p calibration curve. miR-195b Ct values were then extrapolated.

### Heatmap representations

Normalized qPCR data were graphically plotted as heatmaps using Morpheus software (https://software.broadinstitute.org/morpheus/, accessed on 3 March 2020).

### Statistical analyses

For statistical analyses of datasets, unpaired Student’s t-tests were used. Significance levels or *p* values are stated in each corresponding figure legend. *p* < 0.05 was considered statistically significant.

## Results

### Expression pattern of miR-195 during embryonic development

**Figure 1:**
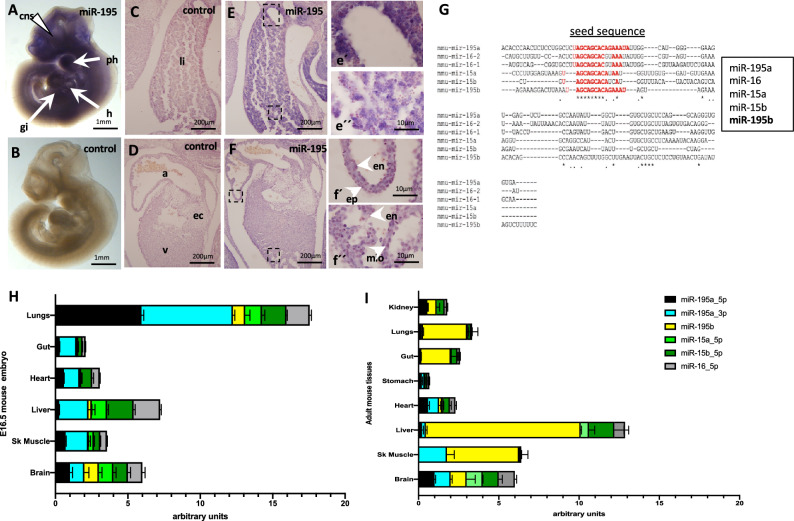
miR-195 expression analyses. In situ hybridization of miR-195 expression (panel **A**, **E** and **F**) and negative controls (**B**, **C** and **D**) in E9.5 (panels **A**, **B**) and E12.5 (panels **C**–**F**) mouse embryos. e′ and e′′ are close ups of panel (**E**). f′ and f′′ are close ups of panel (**F)**. Sequence comparison of the pre-miRNAs of miR-15/miR-16/miR-195 family members (panel **G**), highlighting in red the seed sequence of each mature microRNAs. RT-qPCR analyses of miR-15/miR-16/miR-195 family members in E16 embryonic (panel **H**) and 6 months old adult (panel **I**) mouse tissues. Note that miR-195a, and to a lesser extend miR-195b, are widely expressed in both embryonic and adult tissues. n = 6 in all in situ hybridization experiments. n = 3 in all RT-qPCR experiments.

We analyzed the expression pattern of miR-195 by in situ hybridization at two distinct developmental stages in mouse embryos. Whole-mount in situ hybridization at E10.5, demonstrated a wide expression of miR-195 along the entire embryo, including the central nervous system, the cardiopharyngeal area, the heart and the gastrointestinal tract (Fig. 1A, B). Tissue section analyses in E12.5 embryos demonstrate that expression is observed primarily in the hepatoblasts within the fetal liver (Fig. 1C–F), while within the heart, expression is overtly observed in all three layers, epicardial, myocardial and endocardial cells, with less abundant expression in the endocardial cushions. Overall, these data demonstrate that miR-195 is widely expressed in all embryonic tissues. Taqman assays further validated these findings revealing a ~ fourfold increased levels in liver tissues at 30 days (1.7 pg) as compared to 420 days (0,39 pg).

### Tissue distribution of miR-15/miR-16/miR-195 family members

Since the in situ hybridization probe for miR-195 does not distinguish between distinct isoforms, i.e. miR.195a vs miR-195b, we subsequently analyzed the tissue distribution of different members of the miR-15/16/195 family (Fig. 1G) that allowed us to distinguish between these isoforms, i.e. miR-195a_5p, miR-195a_3p, miR-195b, miR-15a_5p, miR-15b_3p and miR-16_5p at embryonic (E16.5) and adult stages. Embryonic expression of miR-195a_5p is primarily observed in the lungs, followed by the brain, heart and skeletal muscle, with lower expression levels in the gut and liver (Fig. 1H). On the other hand, miR-195a_3p is also robustly expressed in the lungs, followed by the liver, gut, heart and skeletal muscle, with lowest expression levels in the brain (Fig. 1H). Given that both mature microRNAs are derived from the same pre-miR-195a, but they display overt differences in expression in distinct tissues, e.g. skeletal muscle and kidney, these data suggest that additional post-transcriptional mechanisms govern microRNA abundance within the same tissue. miR-195b is expressed in the lungs, brain and liver, with just barely detectable levels in the gut, heart, and skeletal muscle (Fig. 1H). miR-15a_5p is primarily expressed lungs, liver and brain, with almost no detectable levels in gut, heart and skeletal muscle (Fig. 1H). miR-15b_5p is expressed in all tissues analyzed, ranging from those with higher expression levels such as lungs, liver, heart and brain to those with lower expression levels, i.e. gut and skeletal muscle (Fig. 1H). Finally, miR-16_5p is similarly expressed in all tissues, with highest expression in lungs, liver and brain and lower expression levels in gut, heart and skeletal muscle (Fig. 1H). Overall these data demonstrate a wide embryonic distribution of all distinct miR-15/16/195 family members in most of the tissues analyzed.

Subsequently, we also analyzed the expression of the miR-15/16/195 family members in adult tissues. miR-195a_5p displayed enhanced expression in kidney, heart and brain while lower expression levels are detected in lungs, gut, stomach, liver and skeletal muscle (Fig. 1I). On the other hand, miR-195a_3p is enriched in heart, liver, skeletal muscle and brain, while barely detectable expression is observed in kidney lungs, gut and stomach (Fig. 1I). In line with previous results in embryonic tissues, these data suggest that additional post-transcriptional mechanisms govern microRNA abundance within the same tissue. miR-195b is abundantly expressed in liver, lungs, gut, and skeletal muscle, with moderate expression levels in kidney and brain while just barely detectable levels are observed in heart and stomach (Fig. 1I).

miR-15a_5p is primarily expressed in liver and brain, with just barely detected expression in all the other tissues, i.e. kidney, lungs, stomach, heart and skeletal muscle (Fig. 1I). miR-15b_5p displays enhanced expression in kidney, liver and brain with lower expression levels in lungs, gut, stomach, gut and heart while just detectable levels are observed in skeletal muscle (Fig. 1I). Finally, miR-16_5p is prominently expressed in liver and brain, with lower expression levels in kidney, lungs, gut, stomach and heart, an just barely detectable levels in skeletal muscle (Fig. 1I). Overall, these data demonstrate that all distinct miR-15/miR-16/miR-195 family members are expressed in most of the adult tissues analyzed displaying distinct tissue specific expression levels.

### Generation of miR-195b null mice

The functional role of miR-195, particularly miR-195a, has been reported by both gain- and loss-of-function assays^43–45^. However, the role of miR-195b has not been described. We therefore generated miR-195b deficient mice by CRISPR/Cas9 gene editing providing a deletion of $\sim$ 150 bp flanking the miR-195b precursor sequence as depicted in Supplementary Fig. 1A. Founder mice were viable and fertile. To generate distinct miR-195b deficient mice and to minimize the plausible effect of off-targets, founder mice were crossed with wild-type CBA/BL6. miR-195b heterozygous were genotyped and intercrossed, establishing three distinct lines. Mice were bread to adulthood and processed for anatomical and histological examination. No increased mortality was observed in any of the three distinct miR-195b null transgenic mice. Therefore, we selected only one single line for further characterization of miR-195b deficiency.

### miR-195b null mice displayed no overt anatomical or histological alterations

Macroscopic analyses of miR-195b^+/+^, miR-195b^+/−^ and miR-195b^−/−^ mice displayed no gross anatomical significant differences in any major system, including the cardiovascular, gastrointestinal and urogenital systems at different developmental stages, ranging from perinatal stages up to almost 2 years, i.e. 600 days (data not shown). Analyses of the heart to body weight in males and females of miR-195b^+/+^, miR-195b^+/−^ and miR-195b^−/−^ mice displayed no significant differences (Supplementary Fig. 2A). Similarly, no differences were observed in liver/body weight ratio (Supplementary Fig. 2B). We subsequently analyzed at histological levels all major organs of miR-195b^+/+^, miR-195b^+/−^ and miR-195b^−/−^ at five different postnatal stages (30, 60, 120, 240 and 420 postnatal days, respectively). In line with the anatomical analyses, no gross histological alterations were observed, including absence of increased fibrosis in any of the tissues analyzed (Supplementary Fig. 2C), as detailed in the following paragraphs.

### miR-195b deficiency significantly impairs liver homeostasis in elderly mice

**Figure 2:**
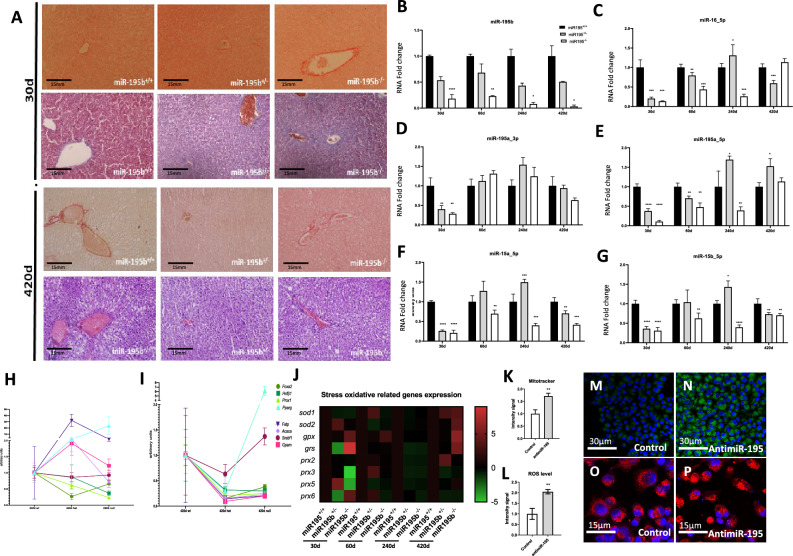
Histological and molecular characterization of miR-195b deficient liver. Hepatic histological sections of miR-195b^+/+^, miR-195b^+/−^ and miR-195b^−/−^ stained with H&E and picrosirius red, corresponding to 30 days and 420 days postnatal livers (panel **A**). Observe that no gross histological differences are observed, including no significant differences in collagen deposition. RT-qPCR analyses of miR-15/miR-16/miR-195 family members in livers corresponding to miR-195b^+/+^, miR-195b^+/−^ and miR-195b^−/−^ mice, respectively, at different postnatal stages (i.e. 30, 60, 240 and 420 days postnatal) (panels **B**–**G**). Observe that miR-195b expression is severely downregulated in miR-195b^+/−^ livers and almost completely absent in miR-195b^−/−^ livers at all stages analyzed, while the rest of the miR-15/miR-16/miR-195 family members display no overt significant up-regulation in miR-195b^+/−^ and miR-195b^−/−^ livers in most stages analyzed, with the exception of a significant upregulation in the liver of miR-195b^+/−^ at 240 days postnatal, but not for miR-195b^−/−^, at any of the stages analyzed. qRT-PCR analyses of *Foxa2, Hnfβ1, Prox1, Pparγ, Fatp, Acaca, Serbf1* and *Gpam* in livers of miR-195b^+/+^, miR-195b^+/−^ and miR-195b^−/−^ mice at two distinct postnatal stages, i.e. 240 days and 420 days (panel **H**–**I**). Observe that a global up-regulation of these hepatic developmental and metabolic markers is observed in miR-195b^+/−^ at 240 days postnatal, but not for miR-195b^−/−^ while at elderly stages, i.e. 420 days, a severe down-regulation of these markers is observed at both miR-195b^+/−^ and miR-195b^−/−^ conditions, with the exception of *Serbf1* and *Foxa2* that are mildly and significantly upregulated in miR-195b^−/−^ but not in miR-195b^+/−^ livers, respectively. RT-qPCR analyses of ROS markers in livers of miR-195b^+/+^, miR-195b^+/−^ and miR-195b^−/−^ mice at four distinct postnatal stages, i.e. 30, 60, 240 and 420 days (panel **J**). Observe that expression levels of *Sod1, Sod2, Gpx* and *Grs* are progressively increased in miR-195b^−/−^ livers with age, while *Prx* genes are severely downregulated at early postnatal stages in miR-195b^+/−^ and miR-195b^−/−^ mice, while at later postnatal stages, there are no significant differences (60 and 240 days) or they become significantly up-regulated (420 days). Mitochondria distribution assessed by Mitotracker labelling (panels **M** and **N**) and quantified (panel **K**) in cell cultures transfected with anti-miR-195 (panel **N**) as compared to controls (panel **M**). Note that anti-miR-195 leads to significant upregulation of the mitochondria labelling. Cell oxidative capacity was measured by CellROX labelling (panels **O**–**P**) and quantified (panel **L**) in cell cultures transfected with anti-miR-195 (panel **P**) as compared to controls (panel **O**). Observe an increase in cellular oxidative capacity after miR-195 inhibition (panel **L**). Normalized qPCR data were graphically plotted as heatmaps using Morpheus software (https://software.broadinstitute.org/morpheus/, accessed on 3 March 2020). All experiments were performed in at least three distinct biological samples. *p < 0.05, ** *p* < 0.01, *** *p* < 0.001, **** *p* < 0.0001.

Several reports have previously demonstrated a regulatory role for miR-195 in cell proliferation, apoptosis and metabolism in different biological settings^45–47^. Since no gross anatomical and histological alterations were observed in hepatic preparations during the lifespan of these mice (Fig. 2A, Supplementary Fig. 2C), we investigated if other miR-15/miR-16/miR-195 family members might be compensating miR-195b loss of function in the liver of miR-195^+/+^, miR-195b^+/−^ and miR-195b^−/−^ mice ranging from 30 to 420 post-natal days. Our data demonstrate that miR-195b was significantly down-regulated in the liver of miR-195b^−/−^ mice to almost no detectable levels, while miR-195b^+/−^ mice displayed a significant but milder down-regulation in all postnatal stages analyzed (Fig. 2B), as expected.

At 30 days, we detected that miR-195a_3p, miR-195a_5p, miR-15a_5p, miR-15b_5p and miR-16_5p were similarly down-regulated as miR-195b (Fig. 2C–G). Curiously, while a moderate up-regulation for miR-16_5p, miR-195a_5p, miR-15a_5p and miR-15b_5p was observed in miR-195b^+/−^ at 240 days but not in miR-195b^−/−^ livers, none of these increases are beyond the level of controls in the rest of postnatal stages and conditions, demonstrating that no compensatory events are occurring at these stages, except for 240 days in miR-195b^+/−^ livers (Fig. 2C–G).

We subsequently analyzed the expression profile of distinct key transcription factors involved in endoderm/liver development such as *Foxa2, Hnfβ1, Prox1* and *Pparγ*, since their expression levels are frequently altered in distinct hepatic pathophysiological conditions^48,49^. Our data demonstrate that *Foxa2, Hnfβ1* and *Prox1* are downregulated at 240 days and 420 days postnatal stages in the liver of miR-195b^+/−^ and miR-195b^−/−^ mice. On the other hand, *Pparγ* is significantly upregulated in miR-195b^+/−^ and miR-195b^−/−^ at 240 days postnatal stage as well as in miR-195b^−/−^ but not in miR-195b^+/−^livers at 420 days postnatal stage (Fig. 2H, I, Supplementary Fig. 3). Thus, these data demonstrated that these transcription factors are severely impaired in the liver of elderly stages in miR-195b^−/−^ mice.

Similarly, RT-qPCR analyses of key elements regulating liver metabolism, i.e. fatty acids, steroids and glycerolipids, such as *Fatp*, *Acaca*, *Srebf1* and *Gpam* displayed no significant differences (*Srebf1* and *Gpam*) or mild upregulation at 240 days postnatal stage in miR-195b^−/−^ mutants, while they were severely down-regulated at elderly stages (420 days), with the exception of *Srebf1* that is barely modified. Overall these data demonstrate a progressive impairment on key developmental and metabolic regulators in the liver of miR-195b deficient mice that are aggravated with aging (Fig. 2H, I, Supplementary Fig. 3), particularly those affecting fatty acids and glycerolipids metabolism.

We subsequently tested if reactive oxidative stress (ROS) markers were impaired in the liver of miR-195b deficient mice, such as those destroying superoxidase radials in distinct subcellular compartments, (*Sod1, Sod2*) and those transferring and/or reducing hydroperoxides (*Gpx, Grs* and peroxiredoxins, *Prx2-Prx6*). Analyses of *Sod1* expression display down-regulation at 30 days (miR-195b^+/−^ and miR-195b^−/−^) while it is up-regulated at 420 days (miR-195b^+/−^ and miR-195b^−/−^) with no significant differences at intermediate stages. *Sod2* only displays significant up-regulation at 30 days (miR-195b^+/−^) and 420 days (miR-195b^−/−^) in line with the similar observations for *Gpx* and *Gsr,* at 30 days on miR-195b^+/−^ and miR-195b^−/−^ and 420 days on miR-195b^−/−^ (Fig. 2J, Supplementary Fig. 4). RT-qPCR analyze of displayed no significant differences for *Prx2* between distinct conditions except at 240 days (miR-195b^+/−^ and miR-195b^−/−^) that is significantly downregulated and at 420 days (miR-195b^−/−^) that is significantly upregulated (miR-195b^−/−^). *Prx3* levels are significantly and decreased at 30 days (miR-195b^−/−^), 240 days (miR-195b^+/−^ and miR-195b^−/−^) and 420 days (miR-195b^+/−^). *Prx5* was significantly upregulated at 30 days in miR-195b^+/−^ but not in miR-195b^−/−^ that is downregulated, as well as at 60 days (miR-195b^+/−^ and miR-195b^−/−^), and 240 days (miR-195b^+/−^ and miR-195b^−/−^) with no significant differences at 420 days (miR-195b^−/−^), while *Prx6* was significantly up-regulated at 30 days (miR-195b^−/−^) and 60 days (miR-195b^+/−^) but down-regulated at 240 days (miR-195b^+/−^) and 420 days (miR-195b^+/−^ and miR-195b^−/−^)(Fig. 2J). Overall these data demonstrate that, although some ROS impairment is observed at mid postnatal stages, severe impairment is mostly observed in the elderly stages (420 days), a period in which most ROS members are consistently up-regulated (i.e. *Sod1, Sod2, Gpx* and *Grs* (Supplementary Fig. 4). To further support the functional role of miR-195 in ROS homeostasis, Hep2G cells were transfected with anti-miR-195 and mitochondrial content (*Mitotracker*) and oxidative status (*CellROX*) were evaluated. Silencing of miR-195 resulted in a significant increase of both markers (Fig. 2K–P), demonstrating the functional role of miR-195 regulating ROS homeostasis.

**Figure 3:**
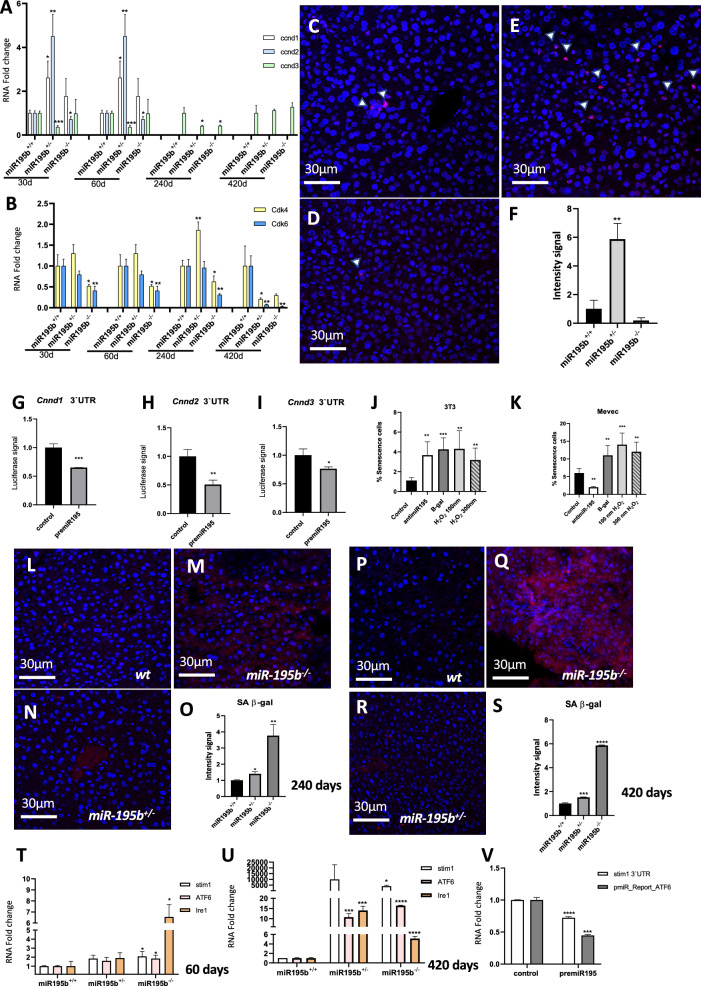
Cell cycle and senescence analyses in miR-195b deficient liver. RT-qPCR analyses of cell cycle markers such as *ccnd1*, *ccnd2*, *ccnd3* (panel **A**), *cdk4* and *cdk6* (panel **B**) in livers of miR-195b^+/+^, miR-195b^+/−^ and miR-195b^−/−^ mice at four distinct postnatal stages, i.e. 30, 60, 240 and 420 days. Note that these cell cycle markers are increased at early postnatal stages in miR-195b^+/−^ and miR-195b^−/−^ mice but are severely downregulated at late postnatal stages. Immunohistochemical analyses of ki67 expression on hepatic histological sections of miR-195b^+/+^ (panel **C**), miR-195b^+/−^ (panel **E**) and miR-195b^−/−^ (panel **D**) mice at 60 days old and their quantitative analyses (panel **F**) demonstrated increased and decreased proliferation in miR-195b^+/−^ and miR-195b^−/−^ mice, respectively, in line with RT-qPCR analyses. Luciferase assays of *ccnd1* 3′UTR (panel **J**), *ccnd2* 3′UTR (panel **K**) and *ccnd3* 3′UTR (panel **L**) demonstrating significant downregulation after miR-195 overexpression in 3T3 fibroblasts. SA-β-galactosidase positive cells in cell cultures of 3T3 fibroblasts (panel **M**) and MEVEC endocardial cells (panel N) transfected with anti-miR-195, β-galactosidase expression vector or treated with 100 mM and 300 nM of H_2_0_2_. Observe the distinct behavior of anti-miR-195 administration in 3T3 fibroblasts (panel **M**) as compared to MEVEC endocardial cells (panel **N**). SA-β-galactosidase positive cells on hepatic histological sections of miR-195b^+/+^ (panel **O**), miR-195b^+/−^ (panel **Q**) and miR-195b^−/−^ (panel **P**) mice and their quantitative analyses (panel **R**) at 240 days, demonstrating increased senescence in miR-195b^−/−^ (panel **P**) mice. SA-β-galactosidase positive cells on hepatic histological sections of miR-195b^+/+^ (panel **S**), miR-195b^+/−^ (panel **U**) and miR-195b^−/−^ (panel **T**) mice and their quantitative analyses (panel **V**) at 420 days, demonstrating increased senescence on miR-195b^−/−^ (panel **T**) mice. RT-qPCR analyses of ER stress markers such as *Stim1, Atf6* and *Ire1* (panels **W**–**X**) in livers of miR-195b^+/+^, miR-195b^+/−^ and miR-195b^−/−^ mice at two distinct postnatal stages, i.e. 60 (panel **W**) and 420 days (panel **X**). Luciferase assays of *Stim1* 3′UTR (panel **Y**) and *Atf6* 3′UTR (panel **Z**) demonstrating significant downregulation after miR-195 overexpression in 3T3 fibroblasts. All experiments were performed in at least three distinct biological samples. *p < 0.05, **p < 0.01, ***p < 0.001, ****p < 0.0001.

Finally, we also analyzed cell cycle regulation, by measuring by RT-qPCR the expression of *Cnnd1, Cnnd2, Cnnd3* cyclins and *Cdk4* and *Cdk6* cyclin-dependent kinases. Our analyses demonstrated that *Cnnd1* and *Cnnd2*, but not *Cnnd3* are significantly upregulated in miR-195b^+/−^ and mildly up-regulated in miR-195b^−/−^ at early postnatal stages, i.e. 30 and 60 days, respectively, while *Cdk4* and *Cdk6* are downregulated (Fig. 3A, B). Surprisingly, at later post-natal stages, *Cnnd1*, *Cnnd2* are not detectable within any experimental condition, *Cnnd3* is decreased at 240 days in miR-195b^+/−^ and miR-195b^−/−^ but not at 420 days, while *Cdk4* and *Cdk6* are significantly downregulated at 420 days not but at 240 days in miR-195b^+/−^ and miR-195b^−/−^ downregulated (Fig. 3A, B). In line with these findings, ki67 labelling in the liver of miR-195b^+/−^ mice is significantly upregulated while it is significantly downregulated in miR-195b^−/−^ mice (Fig. 3C–F). Mechanistically, miR-195b can influence cell cycle progression, as *Ccnd1*, *Ccnd2* and *Ccnd3* are direct targets, as revealed by luciferase assays (Fig. 3G–I).

Additionally, we tested whether cell senescence was influenced by miR-195b deletion. SA-β-galactosidase activity analyses in 240 and 420 day-old livers demonstrated that deletion of miR-195b significantly increased SA-beta-galactosidase expression, both in miR-195b^+/−^ and miR-195b^−/−^ conditions at both postnatal stages (Fig. 3L–S). To further support the plausible role of miR-195b in cell senescence, analyses of SA-β-galactosidase expression were performed in 3T3 fibroblasts and MEVEC endocardial cells treated with antimiR-195. Our data demonstrated that miR-195 inhibition significantly up-regulated SA-β-galactosidase expression to similar levels as H_2_0_2_ administration (Fig. 3J, K), that served as positive control of senescence induction in 3T3 fibroblasts but not in MEVEC cells, supporting a cell-type specific modulation.

Finally, we tested whether markers of endoplasmic reticulum (ER) stress were also altered. RT-qPCR analyses demonstrated that *Stim1*, *Atf6* and *Ire1* were significantly increased in both miR-195b^+/−^ and miR-195b^−/−^ conditions at two distinct developmental stages analyzed, i.e. 60 days (Fig. 3T) and 420 days (Fig. 3U). Mechanistically, impaired expression of these ER makers is directly mediated by miR-195 targeting as revealed by luciferase assays for *Stim1* and *Atf6* 3′UTRs (Fig. 3V).

Overall, these data demonstrate that miR-195b cannot be compensated by other miR-15/16/195 family members in the liver, progressively leading to a progressive molecular dysfunction with age that compromises cell cycle regulation, cell senescence, mitochondrial function and ROS and ER homeostasis, biological processes that are tightly interconnected between each other^50–53^ and if impaired, frequently lead to distinct pathophysiological alterations.

### miR-195b deficiency mildly impairs heart homeostasis

**Figure 4:**
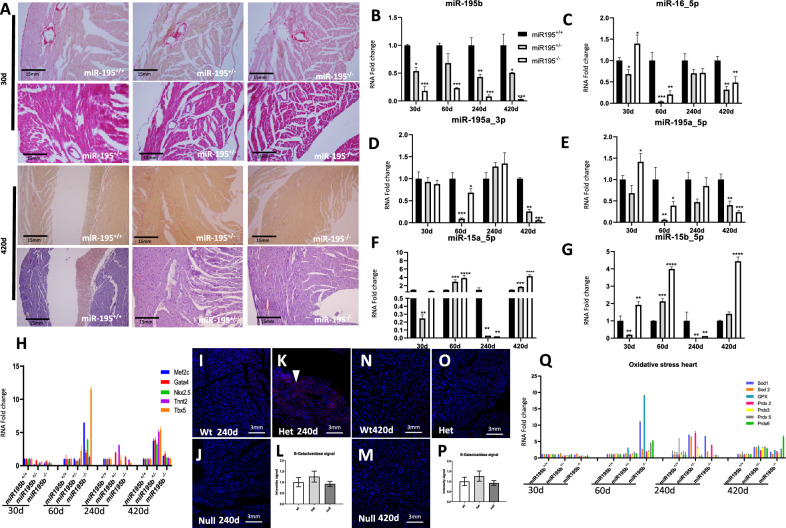
Histological and molecular characterization of miR-195b deficient heart. Cardiac histological sections of miR-195b^+/+^, miR-195b^+/−^ and miR-195b^−/−^ stained with H&E and picrosirius red, corresponding to 30 days and 420 days postnatal livers (panel **A**). Observe that no gross histological differences are observed, including no significant differences in collagen deposition. RT-qPCR analyses of miR-15/miR-16/miR-195 family members in hearts corresponding to miR-195b^+/+^, miR-195b^+/−^ and miR-195b^−/−^ mice, respectively, at different postnatal stages (i.e. 30, 60, 240 and 420 days postnatal) (panels **B**–**F**). Observe that several miR-15/miR-16/miR-195 family members display a significant upregulation in miR-195b^+/−^ and miR-195b^−/−^ hearts in elderly stages analyzed, particularly miR-15a_5p and miR-15b_5p. RT-qPCR analyses of cardiac developmental markers such as *Mef2c, Gata4, Nkx2.5, Tnnt2* and *Tbx5* in hearts of miR-195b^+/+^, miR-195b^+/−^ and miR-195b^−/−^ mice at four distinct postnatal stages, i.e. 30 days to 420 days (panel **G**). Observe that a global upregulation of these cardiac developmental markers is observed in miR-195b^+/−^ and miR-195b^−/−^ hearts from 60 days postnatal onwards, respectively. SA-β-galactosidase positive cells on cardiac histological sections of miR-195b^+/+^ (panel **H**), miR-195b^+/−^ (panel **I**) and miR-195b^−/−^ (panel **J**) mice and their quantitative analyses (panel K) at 240 days, demonstrating no increased senescence on miR-195b^−/−^ (panel **J**) mice. SA-β-galactosidase positive cells on cardiac histological sections of miR-195b^+/+^ (panel **L**), miR-195b^+/−^ (panel **M**) and miR-195b^−/−^ (panel **N**) mice and their quantitative analyses (panel **O**) at 420 days, similarly demonstrating no increased senescence on miR-195b^−/−^ (panel **N**) mice. qRT-PCR analyses of ROS markers in hearts of miR-195b^+/+^, miR-195b^+/−^ and miR-195b^−/−^ mice at four distinct postnatal stages, i.e. 30, 60, 240 and 420 days (panel **P**). Observe that expression of ROS markers are similarly increased in miR-195b^+/−^ and miR-195b^−/−^ hearts with age. All experiments were performed in at least three distinct biological samples. *p < 0.05, **p < 0.01, ***p < 0.001, ****p < 0.0001.

Histological analyses of miR-195b^+/−^ and miR-195b^−/−^ hearts revealed no significant differences as illustrated in Fig. 4A at different postnatal stages (30 and 420 days) as compared to controls (Supplementary Fig. 2C). We subsequently investigated the functional consequences of miR-195b deficiency by monitoring the expression levels of miR-15/miR-16/miR-195 family members in miR-195b^+/+^, miR-195b^+/−^ and miR-195b^−/−^ hearts, spanning from 30 to 420 postnatal days. It is important to highlight that significant upregulation of miR-195a_5p, miR-15b_5p and miR-16_5p is observed in miR-195b^−/−^ hearts at 30 days postnatal. At 60 days, upregulation is observed for miR-15a_5p and miR-15b_5p as well as at 420 days (Fig. 4B–F). Therefore, a sustained up-regulation of distinct miR-15/miR-16/miR-195 family members is observed from early to aged postnatal stages, with the surprising exception of 240 days. Thus, these data demonstrate a compensatory expression of distinct miR-15/miR-16/miR-195 family members in miR-195b^−/−^ hearts, in contrast to the observations reported in miR-195b^−/−^ liver.

We subsequently tested whether distinct cardiac-enriched transcription factors such as *Mef2c, Gata4, Nkx2.5* or *Tbx5* are differentially regulated in miR-195b deficient hearts, as they are frequently deregulated in distinct cardiac pathophysiological conditions^54,55^. Our data demonstrate that these transcription factors are significantly downregulated in miR-195b^+/−^ and miR-195b^−/−^ hearts at 30 days while they are significantly upregulated in miR-195b^+/−^ and miR-195b^−/−^ hearts at 60 days as well as in 240 and 420 days. Curiously, a more prominent upregulation is observed in miR-195b^+/−^ hearts at these later stages (Fig. 4G, Supplementary Fig. 5).

Additionally, we also tested whether cell senescence was influenced by miR-195b deletion in the heart tissues. SA-β-galactosidase analyses in 240 and 420 days postnatal hearts demonstrated a mild but significant increased SA-β-galactosidase expression in miR-195b^+/−^ at 240 days while no significant differences were observed in miR-195b^+/−^ and miR-195b^−/−^ hearts at 420 days (Fig. 4H–O).

Finally, we analyzed whether oxidative stress markers were distinctly regulated in miR-195b^−/−^ hearts during postnatal stages. RT-qPCR analyses of *Sod1, Sod2* and *Gpx* demonstrated no significant differences in early postnatal stages, i.e. 30 days, while at 60 days, 240 days and 420 days significant upregulation was observed in all ROS markers analyzed in miR-195b^+/−^ and miR-195b^−/−^ hearts, respectively. Similarly, the expression of peroxiredoxins (*Prdx2, Prdx3, Prdx5* and *Prxd6*) display no significant differences at postnatal 30 days but are consistently up-regulated in 60, 240 and 420 days postnatal in miR-195b^+/−^ and miR-195b^−/−^ hearts (Fig. 4P, Supplementary Fig. 6).

Overall, these data demonstrate that miR-195b can be partially compensated by other miR-15/16/195 family members in the heart, leading progressively to a mild molecular dysfunction with age that partially compromises cardiac hypertrophy markers and ROS homeostasis but without cell senescence induction.

### miR-195b deficiency is partially rescued by miR-15/miR-16/miR-195 family members in the lungs

**Figure 5:**
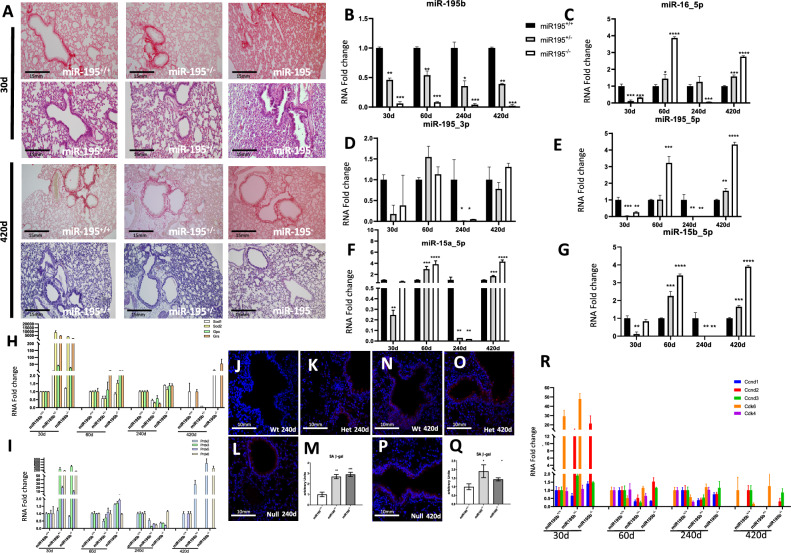
Histological and molecular characterization of miR-195b deficient lung. Lung histological sections of miR-195b^+/+^, miR-195b^+/−^ and miR-195b^−/−^ stained with H&E and picrosirius red, corresponding to 30 days and 420 days postnatal lungs (panel **A**). Observe that no gross histological differences are observed, including no significant differences in collagen deposition. RT-qPCR analyses of miR-15/miR-16/miR-195 family members in lungs corresponding to miR-195b^+/+^, miR-195b^+/−^ and miR-195b^−/−^ mice, respectively, at different postnatal stages (i.e. 30, 60, 240 and 420 days postnatal) (panels **B**–**F**). Observe that several miR-15/miR-16/miR-195 family members display a significant up-regulation in miR-195b^+/−^ and miR-195b^−/−^ lungs in elderly stages analyzed, particularly miR-195a_5p, miR-15b_5p and miR-16_5p. RT-qPCR analyses of ROS markers in lungs of miR-195b^+/+^, miR-195b^+/−^ and miR-195b^−/−^ mice at four distinct postnatal stages, i.e. 30, 60, 240 and 420 days (panels **G**–**H**). Observe that expression of ROS markers are moderately increased in miR-195b^+/−^ and miR-195b^−/−^ hearts with age. SA-β-galactosidase positive cells on lung histological sections of miR-195b^+/+^ (panel **I**), miR-195b^+/−^ (panel **J**) and miR-195b^−/−^ (panel **K**) mice and their quantitative analyses (panel **L**) at 240 days, demonstrating increased senescence on miR-195b^+/−^ (panel **J**) and miR-195b^−/−^ (panel **K**) mice. SA-β-galactosidase positive cells on lung histological sections of miR-195b^+/+^ (panel **M**), miR-195b^+/−^ (panel **N**) and miR-195b^−/−^ (panel **O**) mice and their quantitative analyses (panel **P**) at 420 days, similarly demonstrating no increased senescence on miR-195b^−/−^ (panel **P**) mice. RT-qPCR analyses of cell cycle markers such as *ccnd1, ccnd2, ccnd3, cdk4* and *cdk6* (panel **Q**) in lungs of miR-195b^+/+^, miR-195b^+/−^ and miR-195b^−/−^ mice at four distinct postnatal stages, i.e. 30, 60, 240 and 420 days. Note that *ccnd2* cycle marker is increased only at early postnatal stages in miR-195b^+/−^ and miR-195b^−/−^ mice while most of them are severely downregulated at late postnatal stages (60–420 days postnatal). All experiments were performed in at least three distinct biological samples. *p < 0.05, **p < 0.01, ***p < 0.001, ****p < 0.0001.

In line with previous findings in the liver and heart of miR-195b^−/−^ mice, no significant histological differences were observed in miR-195b^+/−^ and miR-195b^−/−^, as revealed by H&E and picrosirius staining (Fig. 5A; Supplementary Fig. 2C) as compared to controls. We subsequently tested the expression levels of miR-15/miR-16/miR-195 family members in miR-195b^+/+^, miR-195b^+/−^ and miR-195b^−/−^ lungs, spanning from 30 to 420 days postnatal. At 30 days, no significant differences or downregulation of miR-15/miR-16/miR-195 family members was detected in both miR-195b^+/−^ and miR-195b^−/−^ lungs. However, at 60 and 420 days a significant upregulation of miR-195a_5p, miR-15a_5p, miR-15b_5p and miR-16_5p was observed in both miR-195b^+/−^ and miR-195b^−/−^ lungs. Curiously, at 240 days, the expression of these miR-15/miR-16/miR-195 family members was significantly downregulated in both conditions, i.e. miR-195b^+/−^ and miR-195b^−/−^ lungs (Fig. 5B–F). Overall, these data demonstrate that miR-195b deficiency in the lungs is compensated by upregulation of multiple miR-15/miR-16/miR-195 family members in mid-term and aged lungs, in contrast to the observations in liver and heart, where no compensation (liver) or just a mild and limited compensation (heart) is observed.

We subsequently tested if ROS homeostasis is impaired in miR-195b^−/−^ lungs. *Sod1, Sod2, Gpx* and *Gpr* expression is significantly upregulated at early post-natal stages in both miR-195b^+/−^ and miR-195b^−/−^ as compared to control miR-195b^+/+^ at 30 days. *Gpx* and *Gpr*, but not *Sod1* and *Sod2* are also upregulated at 60 days in both conditions (miR-195b^+/−^ and miR-195b^−/−^) while at 240 days significantly differences are only observed in miR-195b^+/−^ displaying, in this case, downregulation. Curiously at 420 days, only *Sod1* and *Grs* are detectable in control miR-195b^+/+^ lungs, and they are significantly upregulated in miR-195b^−/−^ but not in miR-195b^+/−^ mice (Fig. 5G). A similar temporal profile is observed also for *Prdx2, Prdx3, Prdx5* and *Prdx6*. All peroxiredoxins are upregulated in both miR-195b miR-195b^+/−^ and miR-195b^−/−^ lungs as compared to control miR-195b^+/+^ at 30 days, displaying no significant differences or mild downregulation at 60 and 240 days postnatal, respectively. Curiously, *Prdx2* and *Prdx6* are upregulated in miR-195b^+/−^ and miR-195b^−/−^ as compared to control miR-195b^+/+^ at 420 days (Fig. 5H). Overall these data demonstrate that miR-195b deficiency mildly impacts the oxidative stress homeostasis in the lungs.

Additionally, we also tested whether cell senescence was influenced by miR-195b deletion in the lungs. SA-β-galactosidase activity analyses in 240 and 420 days postnatal lungs demonstrated a significant increased SA-β-galactosidase expression in miR-195b^+/−^ and miR-195b^−/−^ at both 240 and 420 days postnatal (Fig. 5I–P).

Finally, we addressed if cell cycle regulation is affected in the postnatal lungs by miR-195b deficiency. Our data demonstrate that *Ccnd2*, but not *Ccnd1* and Ccnd3 are significantly upregulated at early postnatal lung stages (30 days) in both miR-195b^+/−^ and miR-195b^−/−^ as compared to miR-195b^+/+^ controls. On the other hand these cyclins are either not modified or mildly downregulated at 60 days and 240 days, in miR-195b^+/−^ and miR-195b^−/−^ lungs as compared to miR-195b^+/+^ controls, while at 420 days they are barely detectable even in miR-195b^+/+^ controls (Fig. 5Q, Supplementary Fig. 7). On the other hand, cycle-dependent kinases *Cdk6*, but not *Cdk4*, is significantly upregulated in both miR-195b^+/−^ and miR-195b^−/−^ as compared to miR-195b^+/+^ controls at 30 postnatal days. However, none of them are barely modified at 60 and 240 postnatal days in miR-195b^+/−^ and miR-195b^−/−^ as compared to miR-195b^+/+^ controls, while at 420 days only *Cdk6* is detectable displaying a significant downregulation in miR-195b^+/−^ but not in miR-195b^−/−^ lungs (Fig. 5Q, Supplementary Fig. 7). Overall, these data demonstrate that cell cycle regulation is only partially impaired at early lung postnatal development.

Thus, these data demonstrate that miR-195b can be compensated by other miR-15/16/195 family members in the lungs, leading only to a progressive molecular dysfunction with age that mildly compromises cell cycle progression, cell senescence and ROS homeostasis, biological processes that are tightly related^50–53^, as previously mentioned.

### miR-195 modulates miR-15/miR-16/miR-195 family members expression in a tissue-specific manner

**Figure 6:**
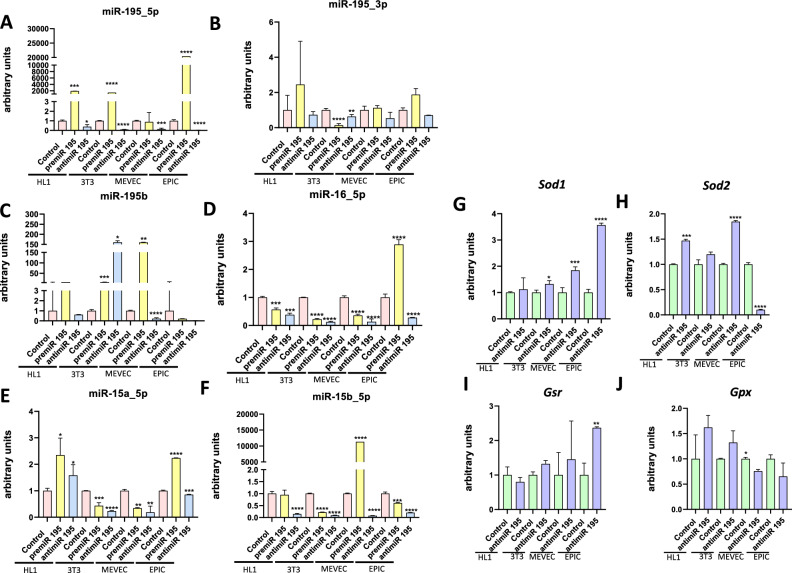
In vitro regulation of miR-15/miR-16/miR-195 family members. RT-qPCR analyses of miR-15/miR-16/miR-195 family members in HL1 cardiomyocytes, 3T3 fibroblasts, MEVEC endocardial and EPIC epicardial cells transfected with pre-miR-195 and anti-miR-195, respectively (panel **A**–**F**). RT-qPCR analyses of ROS markers in HL1 cardiomyocytes, 3T3 fibroblasts, MEVEC endocardial and EPIC epicardial cells transfected with pre-miR-195 and anti-miR-195, respectively (panel **H**–**K**). All experiments were performed in at least three distinct biological samples. *p < 0.05, **p < 0.01, ***p < 0.001, ****p < 0.0001.

Molecular analyses of miR-195b deficiency in heart, lungs and liver demonstrate a tissue-specific mode of action of this microRNA that is particularly evident on the expression levels of distinct members of the miR-195/miR-15/miR16 family. Thus, to further support the tissue-specificity of such co-regulatory mechanisms, we investigated whether miR-195 gain-of-function and loss-of-function in distinct cell types, i.e. HL1 atrial cardiomyocytes, 3T3 fibroblasts, MEVEC endocardial cells and EPIC epicardial cells, can modulate the expression of these microRNAs, i.e. all miR-195/miR-15/miR16 family members. Over-expression of pre-miR-195 leads to up-regulation of miR-195a_5p in all cell types tested, i.e. HL1, 3T3, MEVEC and EPIC cells, while anti-miR-195 treatment lead to significant downregulation (Fig. 6A–F). Curiously, miR-195a-3p was not significantly altered in HL1, MEVEC and EPIC cells after pre-miR-195 gain and loss-of-function treatments, respectively, while it was significantly downregulated in 3T3 fibroblasts in both conditions (Fig. 6A–F). miR-195b was not significantly altered in HL1 and EPIC cells after pre-miR-195 and anti-miR-195 treatment, while it was up-regulated in 3T3 cells in both conditions but it was up-regulated and downregulated in MEVEC after pre-miR-195 and anti-miR-195 treatment, respectively (Fig. 6A–F). miR-15a was upregulated in HL1 cells while it was downregulated in 3T3 and MEVEC cells after both pre-miR-195 and anti-miR-195 administration, respectively. Curiously, in EPIC cells, pre-miR-195 treatment increased miR-15a_5p expression while anti-miR-195 administration lead to miR-15a_3p downregulation (Fig. 6A–F). A similar situation is observed for miR-15b_5p expression as both treatments led to downregulation in 3T3 and EPIC cells, whereas in HL1 cells downregulation was only observed after anti-miR-195 loss-of function while in MEVECs, gain of function leads to upregulation and loss-of-function to downregulation (Fig. 6A–F). Finally, miR-16_5p was downregulated in all cell types analyzed after both miR-195 gain and loss-of-function assays, except in EPIC cells that gain of function led to upregulation and loss-of-function to downregulation (Fig. 6A–F). In sum, these data illustrate that exogenous administration of pre-miR-195 and anti-miR-195 significantly alters the expression of miR-15, miR-16 and miR-195 family members and thus further reinforce the notion that miR-195 expression co-regulates the expression of other microRNAs family members, i.e. miR-15 and miR-16, in a cell-type specific manner.

### miR-195 modulates ROS signaling in a tissue-specific manner

Given the cell type specific role of miR-195 regulating miR-15/miR-16/miR-195 family members, and given that miR-195b deficiently distinctly alters ROS signaling in distinctly organs, we tested whether miR-195 loss-of-function could influence ROS signaling markers in distinct cell types. We demonstrate herein that miR-195 inhibition upregulates *Sod1* expression in all cells types analyzed, i.e. 3T3, MEVEC and EPIC, except HL1 cells (Fig. 6G), while *Sod2* was significantly upregulated in MEVEC and HL1 cells, downregulated in EPIC cells while no significantly differences were detected in 3T3 fibroblasts (Fig. 6H). On the other hand, *Gsr* was only significantly upregulated in EPIC cells (Fig. 6I), while *Gpx* was only significantly downregulated in MEVEC cells, while no significant differences were observed in the other cell types (Fig. 6J). Thus, these data demonstrate that miR-195 can regulate oxidative stress homeostasis in a cell type-specific manner.

## Discussion

Multiple studies have demonstrated the pivotal role of distinct microRNAs in cell homeostasis and their contribution to development of distinct physiopathological conditions. In this context, diverse studies have provided evidence of the functional role of miR-195 in cardiomyogenic^47,56–58^ and hepatic^47,58,59^ cell proliferation and differentiation. Furthermore, multiple evidences reported the involvement of miR-195 in distinct pathological conditions, e.g. lung cancer^60–62^, kidney injury and carcinoma^63–65^, cardiac hypertrophy^66,67^ and fibrosis^68–70^. In this study we provided compelling evidences that miR-195b is distributed in distinct embryonic and adult tissues, supporting thus its involvement in multiple tissues. However, it is important to realize that most studies reported either do not distinguish between the distinct miR-195 isoforms, i.e. miR-195a and miR-195b, or if they do, they are exclusively based on miR-195a (i.e. miR-195a_5p and miR-195a_3p, respectively). Thus, in this study we performed for the first time, miR-195b isoform specific deficient mice by CRISPR/Cas9 gene editing, that resulted in embryonic viable and fertile mice, despite its global and diverse expression in adult tissues. Such an unexpected finding, might be attributed to plausible compensatory mechanisms. We provide herein evidence that miR-195b deficiency distinctly up-regulated other miRNA family members in a tissue-specific and time-dependent manner. In miR-195b deficient liver, only a transitory upregulation of miR-195a_3p, miR-15a_5p and miR-15b_5p is observed in miR-195b^+/−^ mice but not in miR-195b^−/−^ mice, resulting in enhanced disruption of cellular homeostasis as compared to other tissues such as the heart and lungs where sustained temporal upregulation of other miR-195/miR-15/miR-16 family members is observed. Thus, our data support that co-regulatory mechanisms are operative between distinct microRNA family members, as suggested in other biological settings^71–74^, which deserves additional efforts to unravel how are they are mechanistically regulated.

Evidence on the functional role of miR-195 modulating cell cycle progression has been widely documented in distinct biological contexts, including the heart^56,75,76^, the liver^43,46,77,78^ and the lungs^79–83^. Multiple cell cycle regulators such as *Check1*^75^^,^^79^, *Cnne1*^77^, *Cdk4*^77^, *Ccnd1*^84^ and *Ccnd3*^77,81^ have been described as miR-195 direct targets. We provide herein evidences that miR-195b deficiency distinctly impaired cell cycle regulators in distinct tissues. In the liver, *Ccnd2* and *Ccnd3* are highly upregulated in early postnatal stages, in line with our findings by luciferase assays that miR-195 can directly target them. Curiously, such upregulation is no longer observed in aged miR-195b deficient livers, supporting therefore compensatory effects of other miR-15/miR-16/miR-195 family members.

Distinct pathologies are frequently associated with deregulation of metabolic routes and developmental genes, such as hepatocellular carcinoma that downregulates *Foxa2*^48,49^ and cardiac hypertrophy that upregulates *Mef2c*^54,55^. Deregulation of miR-195 expression in liver^85–88^ and cardiac^56,67,76,89^ pathophysiology has been widely documented, however, its functional implication has been scarcely investigated^67,87,89^. In our study we demonstrated that miR-195b deficiency significantly altered key hepatic developmental transcription factors such as *Foxa2* and *Hnfβ1* as well as lipid metabolism in the aged (420 days) deficient liver, a condition that is not observed in early and mid-term (30–240 days) postnatal stages. Importantly, *Foxa2* hepatocyte-specific deletion leads to endoplasmic reticulum stress and impaired live homeostasis^90^. Similarly, deregulation of cardiac developmental genes (*Mef2c, Gata4* and *Nkx2.5*) is also observed in miR-195b deficient hearts. Thus, our data demonstrate that miR-195b deficiency significantly altered cardiac and hepatic cellular homeostasis, a condition that is worsened in elderly stages.

**Figure 7:**
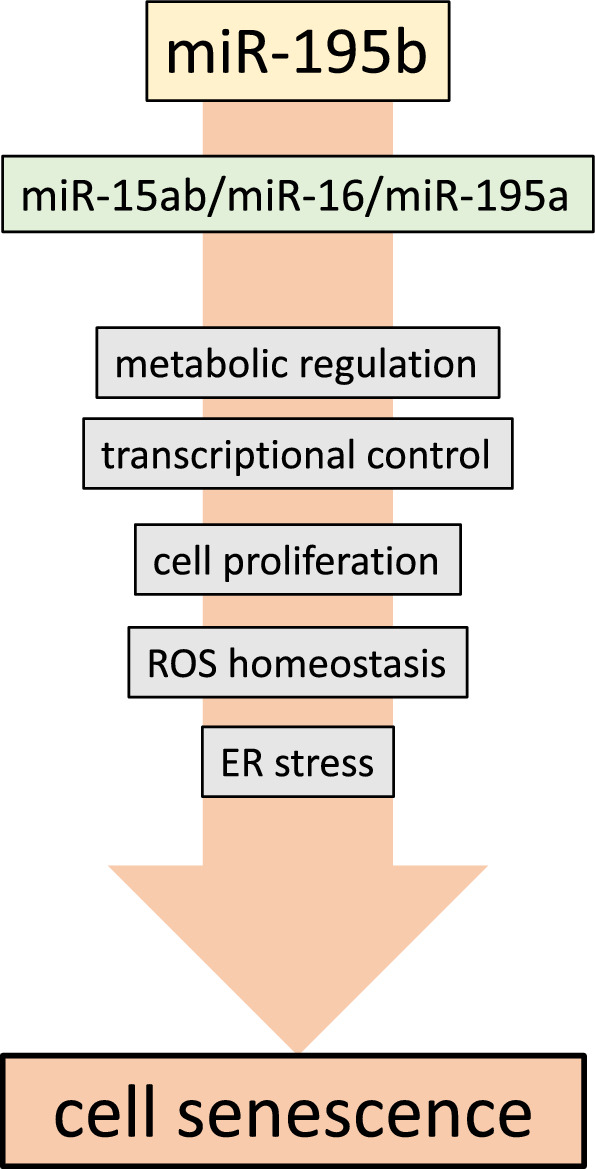
miR-195b working model. Schematic representation of the distinct biological processes modulated by miR-195b. miR-195b deficiency leads to tissue-specific regulation of miR-15/miR-16/miR-195 family members, producing alterations in metabolic regulation, transcriptional control, cell proliferation, ROS homeostasis and ER stress that ultimately lead to cell senescence in the elderly.

While there are previous evidence of the functional role of miR-195 modulating cell proliferation and metabolism, there is scarce information on its plausible role in other essential cellular processes such as redox homeostasis and cellular senescence^91,92^, even if they are intimately co-regulated^50–53^. In our study, we provide compelling in vitro and in vivo evidences that miR-195 deficiency significantly impairs reactive oxygen stress related gene expression and cell senescence in a tissue-specific and stage-dependent manner. It is important to realize that in those tissues where limited miR-15/16/miR195 family members compensation is occurring, i.e. liver, there is increased cellular senescence already at early postnatal stages, while in those with partial compensation, i.e. heart and lungs, there is milder (lungs) or no (heart) cell senescence. Furthermore, we demonstrated that ER stress markers are impaired in the liver in miR-195b deficient mice. Additionally, we demonstrated that *Stim1* and *Atf6* are direct targets of miR-195, as revealed by luciferase assays. Thus, these data support that multiple pathways impair cellular homeostasis in miR-195b deficient mice (Fig. 7).

In summary, we have generated by CRISPR/Cas9 gene editing miR-195b deficient mice that are embryonic viable an fertile but that displayed severe deregulation of key cellular homeostatic processes in the elderly. Importantly, cellular homeostatic dysfunction is selectively affects only in miR-195b deficient liver as compared to miR-195b deficient hearts and lungs. We also demonstrated in vitro and in vivo that such tissue- and stage-specific dysfunction might be attributed to compensatory mechanisms of miR-15/miR-16/miR-195 family members in absence of miR-195b expression. In sum, our data demonstrated that miR-195b is dispensable for embryonic development and adulthood but is required for cellular homeostasis in the elderly.

### Supplementary Information

Supplementary Legends.

Supplementary Figure S1.

Supplementary Figure S2.

Supplementary Figure S3.

Supplementary Figure S4.

Supplementary Figure S5.

Supplementary Figure S6.

Supplementary Figure S7.

Supplementary Figure S8.

Supplementary Table S1.

## Data Availability

Genotype sequencing data are available upon request to the corresponding author.

## References

[1] Hombach S, Kretz M (2016). Non-coding RNAs: Classification, biology and functioning. Adv. Exp. Med. Biol..

[2] Ha M, Kim VN (2014). Regulation of microRNA biogenesis. Nat. Rev. Mol. Cell Biol..

[3] Bartel DP (2018). Metazoan MicroRNAs. Cell.

[4] van Solingen C, Bijkerk R, de Boer HC, Rabelink TJ, van Zonneveld AJ (2015). The role of microRNA-126 in vascular homeostasis. Curr. Vasc. Pharmacol..

[5] Aryal B, Singh AK, Rotllan N, Price N, Fernández-Hernando C (2017). MicroRNAs and lipid metabolism. Curr. Opin. Lipidol..

[6] Dos Santos JAC, Veras ASC, Batista VRG, Tavares MEA, Correia RR, Suggett CB, Teixeira GR (2022). Physical exercise and the functions of microRNAs. Life Sci..

[7] Zhang N, Hu X, Du Y, Du J (2021). The role of miRNAs in colorectal cancer progression and chemoradiotherapy. Biomed. Pharmacother..

[8] Jung H, Kim JS, Lee KH, Tizaoui K, Terrazzino S, Cargnin S, Smith L, Koyanagi A, Jacob L, Li H, Hong SH, Yon DK, Lee SW, Kim MS, Wasuwanich P, Karnsakul W, Shin JI, Kronbichler A (2021). Roles of microRNAs in inflammatory bowel disease. Int. J. Biol. Sci..

[9] Liu Y, Song JW, Lin JY, Miao R, Zhong JC (2020). Roles of MicroRNA-122 in cardiovascular fibrosis and related diseases. Cardiovasc. Toxicol..

[10] Wojciechowska A, Braniewska A, Kozar-Kamińska K (2017). MicroRNA in cardiovascular biology and disease. Adv. Clin. Exp. Med..

[11] Zhou SS, Jin JP, Wang JQ, Zhang ZG, Freedman JH, Zheng Y, Cai L (2018). miRNAS in cardiovascular diseases: Potential biomarkers, therapeutic targets and challenges. Acta Pharmacol. Sin..

[12] DeVeale B, Swindlehurst-Chan J, Blelloch R (2021). The roles of microRNAs in mouse development. Nat. Rev. Genet..

[13] Wystub K, Besser J, Bachmann A, Boettger T, Braun T (2013). miR-1/133a clusters cooperatively specify the cardiomyogenic lineage by adjustment of myocardin levels during embryonic heart development. PLoS Genet..

[14] Fish JE, Santoro MM, Morton SU, Yu S, Yeh RF, Wythe JD, Ivey KN, Bruneau BG, Stainier DY, Srivastava D (2008). miR-126 regulates angiogenic signaling and vascular integrity. Dev. Cell..

[15] Coolen M, Katz S, Bally-Cuif L (2013). miR-9: a versatile regulator of neurogenesis. Front. Cell Neurosci..

[16] Latreille M, Hausser J, Stützer I, Zhang Q, Hastoy B, Gargani S, Kerr-Conte J, Pattou F, Zavolan M, Esguerra JL, Eliasson L, Rülicke T, Rorsman P, Stoffel M (2014). MicroRNA-7a regulates pancreatic β cell function. J. Cli.n Invest..

[17] Gurha P, Abreu-Goodger C, Wang T, Ramirez MO, Drumond AL, van Dongen S, Chen Y, Bartonicek N, Enright AJ, Lee B, Kelm RJ, Reddy AK, Taffet GE, Bradley A, Wehrens XH, Entman ML, Rodriguez A (2012). Targeted deletion of microRNA-22 promotes stress-induced cardiac dilation and contractile dysfunction. Circulation.

[18] Nagosa S, Leesch F, Putin D, Bhattacharya S, Altshuler A, Serror L, Amitai-Lange A, Nasser W, Aberdam E, Rouleau M, Tattikota SG, Poy MN, Aberdam D, Shalom-Feuerstein R (2017). microRNA-184 induces a commitment switch to epidermal differentiation. Stem Cell Rep..

[19] Gagnon JD, Kageyama R, Shehata HM, Fassett MS, Mar DJ, Wigton EJ, Johansson K, Litterman AJ, Odorizzi P, Simeonov D, Laidlaw BJ, Panduro M, Patel S, Jeker LT, Feeney ME, McManus MT, Marson A, Matloubian M, Sanjabi S, Ansel KM (2019). miR-15/16 restrain memory T cell differentiation, cell cycle, and survival. Cell Rep..

[20] Janaki Ramaiah M, Lavanya A, Honarpisheh M, Zarea M, Bhadra U, Bhadra MP (2014). MiR-15/16 complex targets p70S6 kinase 1 and controls cell proliferation in MDA-MB-231 breast cancer cells. Gene..

[21] Ji T, Feng W, Zhang X, Zang K, Zhu X, Shang F (2020). HDAC inhibitors promote pancreatic stellate cell apoptosis and relieve pancreatic fibrosis by upregulating miR-15/16 in chronic pancreatitis. Hum. Cell..

[22] Cimmino, A., Calin, G. A., Fabbri, M., Iorio, M. V., Ferracin, M., Shimizu, M., Wojcik, S. E., Aqeilan, R. I., Zupo, S., Dono, M., Rassenti, L., Alder, H., Volinia, S., Liu, C. G., Kipps, T. J., Negrini, M., Croce, C. M. miR-15 and miR-16 induce apoptosis by targeting BCL2. *Proc. Natl. Acad. Sci. U. S. A. *102(39):13944–13949 (2005). 10.1073/pnas.0506654102. Epub 2005 Sep 15. Erratum in: Proc Natl Acad Sci U S A. 2006 Feb 14;103(7):2464. PMID: 16166262; PMCID: PMC1236577. 10.1073/pnas.0506654102 PMC1236577 16166262

[23] Arora S, Singh P, Tabassum G, Dohare R, Syed MA (2022). miR-16-5p regulates aerobic glycolysis and tumorigenesis of NSCLC cells via LDH-A/lactate/NF-κB signaling. Life Sci..

[24] Porrello ER, Mahmoud AI, Simpson E, Johnson BA, Grinsfelder D, Canseco D, Mammen PP, Rothermel BA, Olson EN, Sadek HA (2013). Regulation of neonatal and adult mammalian heart regeneration by the miR-15 family. Proc. Natl. Acad. Sci. U. S. A..

[25] Tijsen AJ, van der Made I, van den Hoogenhof MM, Wijnen WJ, van Deel ED, de Groot NE, Alekseev S, Fluiter K, Schroen B, Goumans MJ, van der Velden J, Duncker DJ, Pinto YM, Creemers EE (2014). The microRNA-15 family inhibits the TGFβ-pathway in the heart. Cardiovasc. Res..

[26] Ma L, Liu J, Xiao E, Ning H, Li K, Shang J, Kang Y (2021). MiR-15b and miR-16 suppress TGF-β1-induced proliferation and fibrogenesis by regulating LOXL1 in hepatic stellate cells. Life Sci..

[27] An F, Gong B, Wang H, Yu D, Zhao G, Lin L, Tang W, Yu H, Bao S, Xie Q (2012). miR-15b and miR-16 regulate TNF mediated hepatocyte apoptosis via BCL2 in acute liver failure. Apoptosis.

[28] Han SH, Han JH, Chun WJ, Lee SS, Kim HS, Lee JW (2021). Nobiletin inhibits non-small-cell lung cancer by inactivating WNT *β* -catenin signaling through downregulating miR-15-5p. Evid. Based Complement. Alternat. Med..

[29] Xiong Y, Feng Y, Zhao J, Lei J, Qiao T, Zhou Y, Lu Q, Jiang T, Jia L, Han Y (2021). TFAP2A potentiates lung adenocarcinoma metastasis by a novel miR-16 family/TFAP2A/PSG9/TGF-β signaling pathway. Cell Death Dis..

[30] Li Z, Jiang W, Wu G, Ju X, Wang Y, Liu W (2018). miR-16 inhibits hyperoxia-induced cell apoptosis in human alveolar epithelial cells. Mol. Med. Rep..

[31] Moreno-Mateos MA, Vejnar CE, Beaudoin JD, Fernandez JP, Mis EK, Khokha MK (2015). CRISPRscan: Designing highly efficient sgRNAs for CRISPR-Cas9 targeting in vivo. Nat. Methods..

[32] Jinek M, Chylinski K, Fonfara I, Hauer M, Doudna JA, Charpentier E (2012). A programmable dual-RNA-guided DNA endonuclease in adaptive bacterial immunity. Science.

[33] Garcia-Padilla C, Dueñas A, Franco D, Garcia-Lopez V, Aranega A, Garcia-Martinez V, Lopez-Sanchez C (2022). Dynamic MicroRNA expression profiles during embryonic development provide novel insights into cardiac *Sinus Venosus *inflow tract differentiation. Front. Cell Dev. Biol..

[34] Franco D, Markman MM, Wagenaar GT, Ya J, Lamers WH, Moorman AF (1999). Myosin light chain 2a and 2v identifies the embryonic outflow tract myocardium in the developing rodent heart. Anat. Rec..

[35] Lopez-Sanchez C, Franco D, Bonet F, Garcia-Lopez V, Aranega A, Garcia-Martinez V (2015). Negative Fgf8-Bmp2 feed-back is regulated by miR-130 during early cardiac specification. Dev. Biol..

[36] Claycomb WC, Lanson NA, Stallworth BS, Egeland DB, Delcarpio JB, Bahinski A, Izzo NJ (1998). HL-1 cells: A cardiac muscle cell line that contracts and retains phenotypic characteristics of the adult cardiomyocyte. Proc. Natl. Acad. Sci. U. S. A..

[37] D'Amato G, Luxán G, del Monte-Nieto G, Martínez-Poveda B, Torroja C, Walter W, Bochter MS, Benedito R, Cole S, Martinez F, Hadjantonakis AK, Uemura A, Jiménez-Borreguero LJ, de la Pompa JL (2016). Sequential Notch activation regulates ventricular chamber development. Nat. Cell Biol..

[38] Ruiz-Villalba A, Ziogas A, Ehrbar M, Pérez-Pomares JM (2013). Characterization of epicardial-derived cardiac interstitial cells: differentiation and mobilization of heart fibroblast progenitors. PLoS One.

[39] Debacq-Chainiaux F, Erusalimsky JD, Campisi J, Toussaint O (2009). Protocols to detect senescence-associated beta-galactosidase (SA-betagal) activity, a biomarker of senescent cells in culture and in vivo. Nat. Protoc..

[40] Daimi H, Lozano-Velasco E, Haj-Khelil A, Chibani JB, Barana A, Amorós I, de la Fuente MG, Caballero R, Aranega A, Franco D (2015). Regulation of SCN5A by microRNAs: miR-219 modulates SCN5A transcript expression and the effects of flecainide intoxication in mice. Heart Rhythm..

[41] Livak KJ, Schmittgen TD (2001). Analysis of relative gene expression data using real-time quantitative PCR and the 2(-Delta Delta C(T)) Method. Methods..

[42] Lozano-Velasco E, Vallejo D, Esteban FJ, Doherty C, Hernández-Torres F, Franco D, Aránega AE (2015). A Pitx2-MicroRNA pathway modulates cell proliferation in myoblasts and skeletal-muscle satellite cells and promotes their commitment to a myogenic cell fate. Mol. Cell Biol..

[43] Xu T, Zhu Y, Xiong Y, Ge YY, Yun JP, Zhuang SM (2009). MicroRNA-195 suppresses tumorigenicity and regulates G1/S transition of human hepatocellular carcinoma cells. Hepatology..

[44] Zheng D, Ma J, Yu Y, Li M, Ni R, Wang G, Chen R, Li J, Fan GC, Lacefield JC, Peng T (2015). Silencing of miR-195 reduces diabetic cardiomyopathy in C57BL/6 mice. Diabetologia..

[45] Yan JJ, Chang Y, Zhang YN, Lin JS, He XX, Huang HJ (2017). miR-195 inhibits cell proliferation via targeting AEG-1 in hepatocellular carcinoma. Oncol. Lett..

[46] Shi M, Lv X, Zhu M, Dong Y, Hu L, Qian Y, Fan C, Tian N (2022). HMGA1 promotes hepatocellular carcinoma proliferation, migration, and regulates cell cycle via miR-195-5p. Anticancer Drugs.

[47] Wang A, Bu FT, Li JJ, Zhang YF, Jia PC, You HM, Wu S, Wu YY, Zhu S, Huang C, Li J (2022). MicroRNA-195-3p promotes hepatic stellate cell activation and liver fibrosis by suppressing PTEN expression. Toxicol. Lett..

[48] Xu L, Hui L, Wang S, Gong J, Jin Y, Wang Y, Ji Y, Wu X, Han Z, Hu G (2001). Expression profiling suggested a regulatory role of liver-enriched transcription factors in human hepatocellular carcinoma. Cancer Res..

[49] Chang TM, Hung WC (2012). Transcriptional repression of TWIST1 gene by Prospero-related homeobox 1 inhibits invasiveness of hepatocellular carcinoma cells. FEBS Lett..

[50] Miwa S, Kashyap S, Chini E, von Zglinicki T (2022). Mitochondrial dysfunction in cell senescence and aging. J. Clin. Invest..

[51] Ogrodnik M (2021). Cellular aging beyond cellular senescence: Markers of senescence prior to cell cycle arrest in vitro and in vivo. Aging Cell..

[52] Kudryavtseva AV, Krasnov GS, Dmitriev AA, Alekseev BY, Kardymon OL, Sadritdinova AF, Fedorova MS, Pokrovsky AV, Melnikova NV, Kaprin AD, Moskalev AA, Snezhkina AV (2016). Mitochondrial dysfunction and oxidative stress in aging and cancer. Oncotarget..

[53] Cheng H, Gang X, He G, Liu Y, Wang Y, Zhao X, Wang G (2020). The molecular mechanisms underlying mitochondria-associated endoplasmic reticulum membrane-induced insulin resistance. Front. Endocrinol..

[54] Kolodziejczyk SM, Wang L, Balazsi K, DeRepentigny Y, Kothary R, Megeney LA (1999). MEF2 is upregulated during cardiac hypertrophy and is required for normal post-natal growth of the myocardium. Curr. Biol..

[55] Azakie A, Fineman JR, He Y (2006). Myocardial transcription factors are modulated during pathologic cardiac hypertrophy in vivo. J. Thorac. Cardiovasc. Surg..

[56] Porrello ER, Johnson BA, Aurora AB, Simpson E, Nam YJ, Matkovich SJ, Dorn GW, van Rooij E, Olson EN (2011). MiR-15 family regulates postnatal mitotic arrest of cardiomyocytes. Circ. Res..

[57] Dueñas A, Expósito A, Muñoz MDM, de Manuel MJ, Cámara-Morales A, Serrano-Osorio F, García-Padilla C, Hernández-Torres F, Domínguez JN, Aránega A, Franco D (2020). MiR-195 enhances cardiomyogenic differentiation of the proepicardium/septum transversum by Smurf1 and Foxp1 modulation. Sci. Rep..

[58] Sekiya Y, Ogawa T, Iizuka M, Yoshizato K, Ikeda K, Kawada N (2011). Down-regulation of cyclin E1 expression by microRNA-195 accounts for interferon-β-induced inhibition of hepatic stellate cell proliferation. J. Cell Physiol..

[59] Wang R, Fu T, You K, Li S, Zhao N, Yang J, Zhuang SM (2018). Identification of a TGF-β-miR-195 positive feedback loop in hepatocytes and its deregulation in hepatoma cells. FASEB J..

[60] Long Z, Wang Y (2020). miR-195-5p suppresses lung cancer cell proliferation, migration, and invasion via FOXK1. Technol. Cancer Res. Treat..

[61] Chae DK, Park J, Cho M, Ban E, Jang M, Yoo YS, Kim EE, Baik JH, Song EJ (2019). MiR-195 and miR-497 suppress tumorigenesis in lung cancer by inhibiting SMURF2-induced TGF-β receptor I ubiquitination. Mol. Oncol..

[62] Liu H, Chen Y, Li Y, Li C, Qin T, Bai M, Zhang Z, Jia R, Su Y, Wang C (2019). miR-195 suppresses metastasis and angiogenesis of squamous cell lung cancer by inhibiting the expression of VEGF. Mol. Med. Rep..

[63] Xu Y, Jiang W, Zhong L, Li H, Bai L, Chen X, Lin Y, Zheng D (2020). miR-195-5p alleviates acute kidney injury through repression of inflammation and oxidative stress by targeting vascular endothelial growth factor A. Aging.

[64] Chen YQ, Wang XX, Yao XM, Zhang DL, Yang XF, Tian SF, Wang NS (2012). Abated microRNA-195 expression protected mesangial cells from apoptosis in early diabetic renal injury in mice. J. Nephrol..

[65] Wang K, Sun Y, Tao W, Fei X, Chang C (2017). Androgen receptor (AR) promotes clear cell renal cell carcinoma (ccRCC) migration and invasion via altering the circHIAT1/miR-195-5p/29a-3p/29c-3p/CDC42 signals. Cancer Lett..

[66] Xuan, L., Zhu, Y., Liu, Y., Yang, H., Wang, S., Li, Q., Yang, C., Jiao, L., Zhang, Y., Yang, B., Sun, L. Up-regulation of miR-195 contributes to cardiac hypertrophy-induced arrhythmia by targeting calcium and potassium channels. *J. Cell Mol. Med. *24(14), 7991–8005 (2020). 10.1111/jcmm.15431. Epub 2020 May 28. Erratum in: J Cell Mol Med. 2021 Feb;25(3):1801. PMID: 32468736; PMCID: PMC7348160. 10.1111/jcmm.15431 PMC7348160 32468736

[67] Wang L, Qin D, Shi H, Zhang Y, Li H, Han Q (2019). MiR-195-5p promotes cardiomyocyte hypertrophy by targeting MFN2 and FBXW7. Biomed. Res. Int..

[68] Wang DM, Jin JJ, Tian LM, Zhang Z (2020). MiR-195 promotes myocardial fibrosis in MI rats via targeting TGF-β1/Smad. J. Biol. Regul. Homeost. Agents..

[69] Xu, Q., Lin, X. X., Liu, P., Zhang, W., Tang, K., Zhai, Y. S., Liu, L. J., Mei, W. Y. MiR-195 inhibits myocardial fibrosis in hypertensive rats by regulating TGFβ1-Smad3 signaling pathway. *Eur. Rev. Med. Pharmacol. Sci*. 23(18), 8087–8094 (2019) 10.26355/eurrev_201909_19026. Retraction in: Eur Rev Med Pharmacol Sci. 2020 Aug;24(15):7919. PMID: 31599435. 10.26355/eurrev_201909_19026 31599435

[70] Ding H, Yao J, Xie H, Wang C, Chen J, Wei K, Ji Y, Liu L (2021). MicroRNA-195-5p downregulation inhibits endothelial mesenchymal transition and myocardial fibrosis in diabetic cardiomyopathy by targeting Smad7 and inhibiting transforming growth factor beta 1-smads-snail pathway. Front. Physiol..

[71] Song R, Catchpoole DR, Kennedy PJ, Li J (2015). Identification of lung cancer miRNA-miRNA co-regulation networks through a progressive data refining approach. J. Theor. Biol..

[72] Davis JA, Saunders SJ, Mann M, Backofen R (2017). Combinatorial ensemble miRNA target prediction of co-regulation networks with non-prediction data. Nucl. Acids Res..

[73] Gan L, Denecke B (2017). Co-regulation of microRNAs and transcription factors in cardiomyocyte specific differentiation of murine embryonic stem cells: An aspect from transcriptome analysis. Biochim. Biophys. Acta Gene Regul. Mech..

[74] Wu B, Li C, Zhang P, Yao Q, Wu J, Han J, Liao L, Xu Y, Lin R, Xiao D, Xu L, Li E, Li X (2013). Dissection of miRNA-miRNA interaction in esophageal squamous cell carcinoma. PLoS One.

[75] He JF, Luo YM, Wan XH, Jiang D (2011). Biogenesis of MiRNA-195 and its role in biogenesis, the cell cycle, and apoptosis. J. Biochem. Mol. Toxicol..

[76] Busk PK, Cirera S (2010). MicroRNA profiling in early hypertrophic growth of the left ventricle in rats. Biochem. Biophys. Res. Commun..

[77] Furuta M, Kozaki K, Tanimoto K, Tanaka S, Arii S, Shimamura T, Niida A, Miyano S, Inazawa J (2013). The tumor-suppressive miR-497-195 cluster targets multiple cell-cycle regulators in hepatocellular carcinoma. PLoS One..

[78] Yang Y, Li M, Chang S, Wang L, Song T, Gao L, Hu L, Li Z, Liu L, Yao J, Huang C (2014). MicroRNA-195 acts as a tumor suppressor by directly targeting Wnt3a in HepG2 hepatocellular carcinoma cells. Mol. Med. Rep..

[79] Liu B, Qu J, Xu F, Guo Y, Wang Y, Yu H, Qian B (2015). MiR-195 suppresses non-small cell lung cancer by targeting CHEK1. Oncotarget..

[80] Luo J, Pan J, Jin Y, Li M, Chen M (2019). MiR-195-5p inhibits proliferation and induces apoptosis of non-small cell lung cancer cells by targeting CEP55. OncoTargets Ther..

[81] Wang D, Chen Y, Song X, Wu S, Zhang N, Zheng F, Yang G (2022). LncRNA *OXCT1-AS1* promotes the proliferation of non-small cell lung cancer cells by targeting the <i>miR-195/CCNE1</i> axis. Transl. Cancer Res..

[82] Xi J, Xi Y, Zhang Z, Hao Y, Wu F, Bian B, Hao G, Li W, Zhang S (2021). Hsa_circ_0060937 accelerates non-small cell lung cancer progression via modulating miR-195-5p/HMGB3 pathway. Cell Cycle.

[83] Yu X, Zhang Y, Cavazos D, Ma X, Zhao Z, Du L, Pertsemlidis A (2018). miR-195 targets cyclin D3 and survivin to modulate the tumorigenesis of non-small cell lung cancer. Cell Death Dis..

[84] Xu T, Zhu Y, Xiong Y, Ge YY, Yun JP, Zhuang SM (2009). MicroRNA-195 suppresses tumorigenicity and regulates G1/S transition of human hepatocellular carcinoma cells. Hepatology.

[85] Vulf M, Shunkina D, Komar A, Bograya M, Zatolokin P, Kirienkova E, Gazatova N, Kozlov I, Litvinova L (2021). Analysis of miRNAs profiles in serum of patients with steatosis and steatohepatitis. Front. Cell Dev. Biol..

[86] Lendvai G, Szekerczés T, Gyöngyösi B, Schlachter K, Kontsek E, Pesti A, Patonai A, Werling K, Kovalszky I, Schaff Z, Kiss A (2019). MicroRNA expression in focal nodular hyperplasia in comparison with cirrhosis and hepatocellular carcinoma. Pathol. Oncol. Res..

[87] Hu WY, Wei HY, Li KM, Wang RB, Xu XQ, Feng R (2020). LINC00511 as a ceRNA promotes cell malignant behaviors and correlates with prognosis of hepatocellular carcinoma patients by modulating miR-195/EYA1 axis. Biomed. Pharmacother..

[88] Zhu HR, Huang RZ, Yu XN, Shi X, Bilegsaikhan E, Guo HY, Song GQ, Weng SQ, Dong L, Janssen HLA, Shen XZ, Zhu JM (2018). Microarray expression profiling of microRNAs reveals potential biomarkers for hepatocellular carcinoma. Tohoku J. Exp. Med..

[89] Chen H, Untiveros GM, McKee LA, Perez J, Li J, Antin PB, Konhilas JP (2012). Micro-RNA-195 and -451 regulate the LKB1/AMPK signaling axis by targeting MO25. PLoS One.

[90] Bochkis IM, Rubins NE, White P, Furth EE, Friedman JR, Kaestner KH (2008). Hepatocyte-specific ablation of Foxa2 alters bile acid homeostasis and results in endoplasmic reticulum stress. Nat. Med..

[91] Okada M, Kim HW, Matsu-ura K, Wang YG, Xu M, Ashraf M (2016). Abrogation of age-induced MicroRNA-195 rejuvenates the senescent mesenchymal stem cells by reactivating telomerase. Stem Cells..

[92] Kondo H, Kim HW, Wang L, Okada M, Paul C, Millard RW, Wang Y (2016). Blockade of senescence-associated microRNA-195 in aged skeletal muscle cells facilitates reprogramming to produce induced pluripotent stem cells. Aging Cell..

