# Supplementary material for: miR-195b is required for proper cellular homeostasis in the elderly
Source: Sci Rep. 2024 Jan 8;14:810. doi: 10.1038/s41598-024-51256-8 (PMC10774362; doi:10.1038/s41598-024-51256-8)
Supplement: Supplementary file 1 — Supplementary Legends. [file 41598_2024_51256_MOESM1_ESM.pdf]

Supplementary Table and Figure legends

Supplementary Table 1. List of mRNA primer sequences used for RT-qPCR analyses. List of primer sequences used for 3´UTR cloning and subsequent luciferase assays. List of microRNAs primer sequences used for RT-qPCR analyses

Supplementary Figure 1. A. Schematic representation of the miR-195b deletion gene editing strategy. sgRNA guides were designed flanking the miR-195b precursor sequence, resulting in a 400-500 fragment deletion. **B.** PCR analyses of FO founder mice with distinct miR-195b deletion. Red arrows delineate those mice selected for cloning, sequencing and subsequently for the generation of three distinct miR-195b deficient mouse lines. **C.** PCR analyses of miR-195b$^{+/+}$, miR-195b$^{+/-}$ and miR-195b$^{-/-}$ mice.

Supplementary Figure 2. A. Schematic representation of the heart/body weight ratio in miR-195 deficient mice (miR-195b$^{+/+}$, miR-195b$^{+/-}$ and miR-195b$^{-/-}$) according the three age and sex. **B.** Schematic representation of the liver/body weight ratio in miR-195 deficient mice (miR-195b$^{+/+}$, miR-195b$^{+/-}$ and miR-195b$^{-/-}$). Observe that no significant difference are observed. **C.** Quantitative analyses of the fibrotic components using ImageJ software in liver, heart and lung histological sections stained with picrosirius red. Observe that there are no significant differences.

Supplementary Figure 3. RT-qPCR analyses of liver development and lipid metabolism markers in liver samples of miR-195b$^{+/+}$, miR-195b$^{+/-}$ and miR-195b$^{-/-}$ mice, at two distinct postnatal stages, i.e. 240 and 420 days. *p<0.05, ** p<0.01, *** p<0.001, **** p<0.0001.

Supplementary Figure 4. RT-qPCR analyses of ROS markers in liver samples of miR-195b$^{+/+}$, miR-195b$^{+/-}$ and miR-195b$^{-/-}$ mice, at four distinct postnatal stages, i.e. 30, 60, 240 and 420 days. *p<0.05, ** p<0.01, *** p<0.001, **** p<0.0001.

Supplementary Figure 5. RT-qPCR analyses of cardiomyogenic developmental markers in heart samples of miR-195b$^{+/+}$, miR-195b$^{+/-}$ and miR-195b$^{-/-}$ mice, at four distinct postnatal stages, i.e. 30, 60, 240 and 420 days. *p<0.05, ** p<0.01, *** p<0.001, **** p<0.0001.

Supplementary Figure 6. RT-qPCR analyses of ROS markers in heart samples of miR-195b$^{+/+}$, miR-195b$^{+/-}$ and miR-195b$^{-/-}$ mice, at four distinct postnatal stages, i.e. 30, 60, 240 and 420 days. *p<0.05, ** p<0.01, *** p<0.001, **** p<0.0001.

Supplementary Figure 7. RT-qPCR analyses of cell cycle markers in lung samples of miR-195b$^{+/+}$, miR-195b$^{+/-}$ and miR-195b$^{-/-}$ mice, at four distinct postnatal stages, i.e. 30, 60, 240 and 420 days. *p<0.05, ** p<0.01, *** p<0.001, **** p<0.0001.
