# Supplementary material for: miR-195b is required for proper cellular homeostasis in the elderly
Source: Sci Rep. 2024 Jan 8;14:810. doi: 10.1038/s41598-024-51256-8 (PMC10774362; doi:10.1038/s41598-024-51256-8)
Supplement: Supplementary file 2 — Supplementary Figure S1. [file 41598_2024_51256_MOESM2_ESM.pdf]

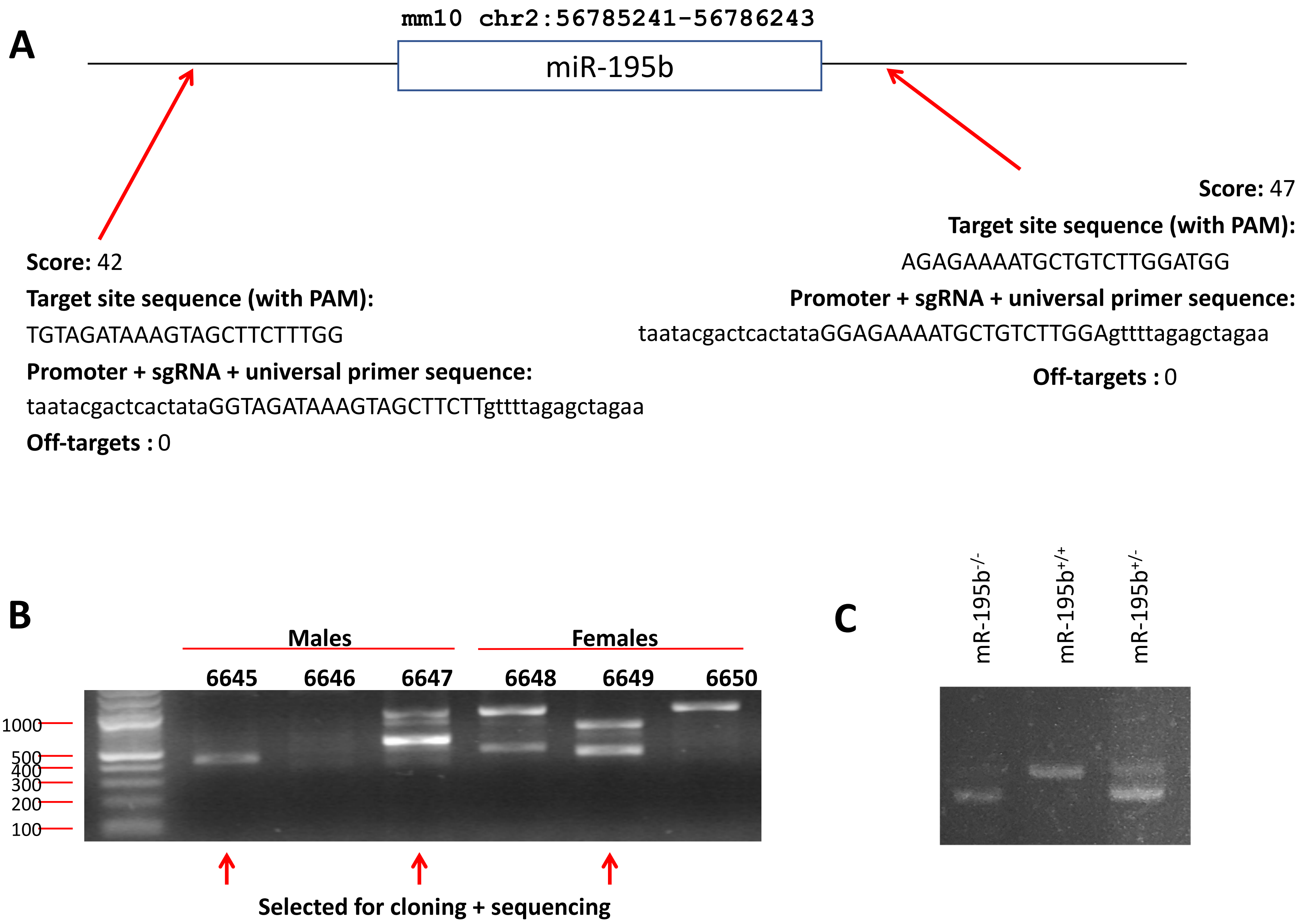
A
mm10 chr2:56785241-56786243
miR-195b
Score: 42
Target site sequence (with PAM):
TGTAGATAAAGTAGCTTCTTTGG
Promoter + sgRNA + universal primer sequence:
taatacgactcactataGGTAGATAAAGTAGCTTCTTgttttagagctagaa
Off-targets : 0
Score: 47
Target site sequence (with PAM):
AGAGAAAATGCTGTCTTGGATGG
Promoter + sgRNA + universal primer sequence:
taatacgactcactataGGAGAAAATGCTGTCTTGGAgttttagagctagaa
Off-targets : 0
B
Males
Females
6645
6646
6647
6648
6649
6650
1000
500
400
300
200
100
Selected for cloning + sequencing
C
mR-195b-/-
mR-195b+/+
mR-195b+/-
