# Supplementary figures and images for: miR-195b is required for proper cellular homeostasis in the elderly
Source: Sci Rep. 2024 Jan 8;14:810. doi: 10.1038/s41598-024-51256-8 (PMC10774362; doi:10.1038/s41598-024-51256-8)

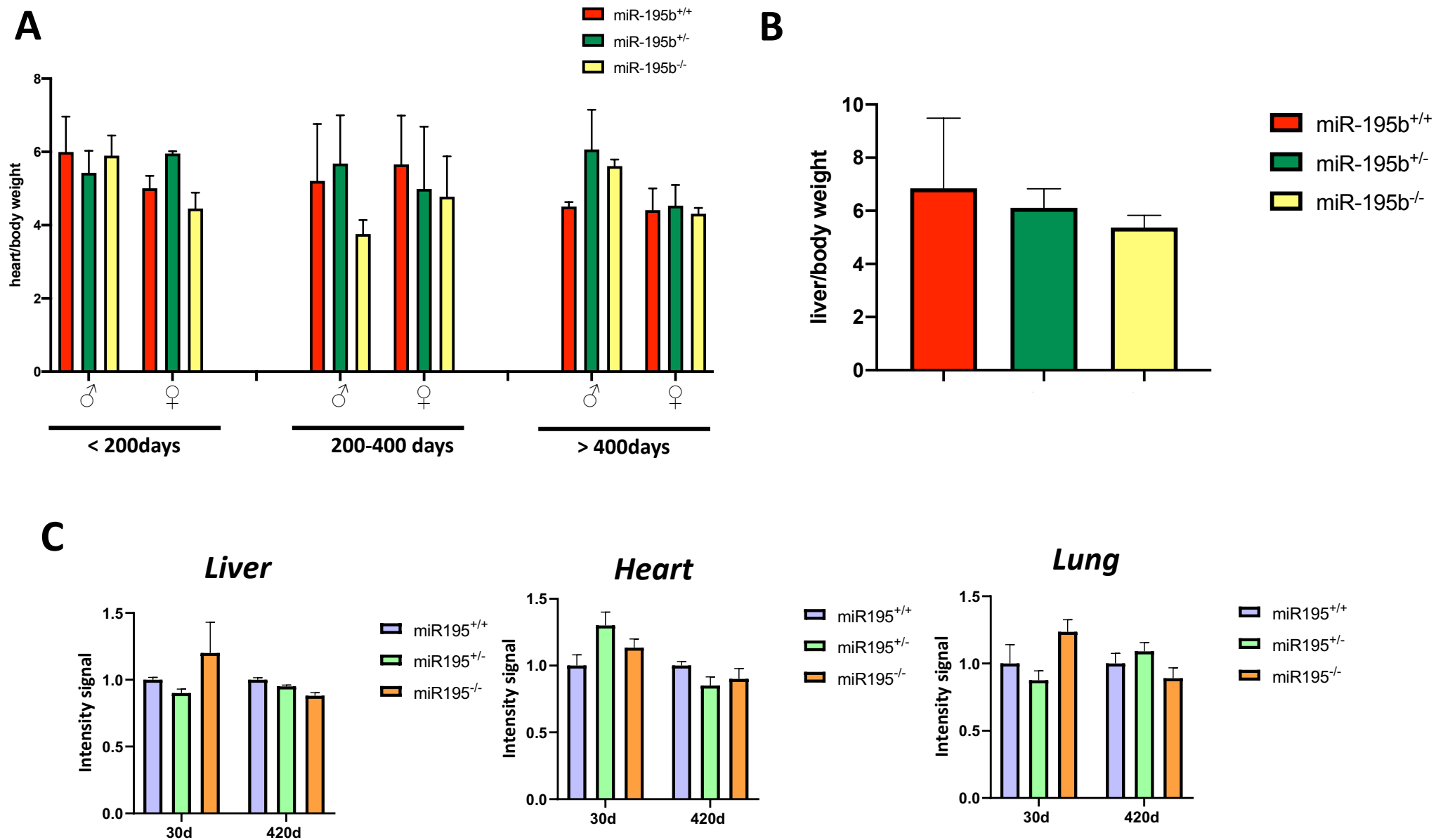

**Supplementary Figure 2. Muñoz-Gallardo et al.**

Supplement: Supplementary file 3 — Supplementary Figure S2. [file 41598_2024_51256_MOESM3_ESM.pdf]

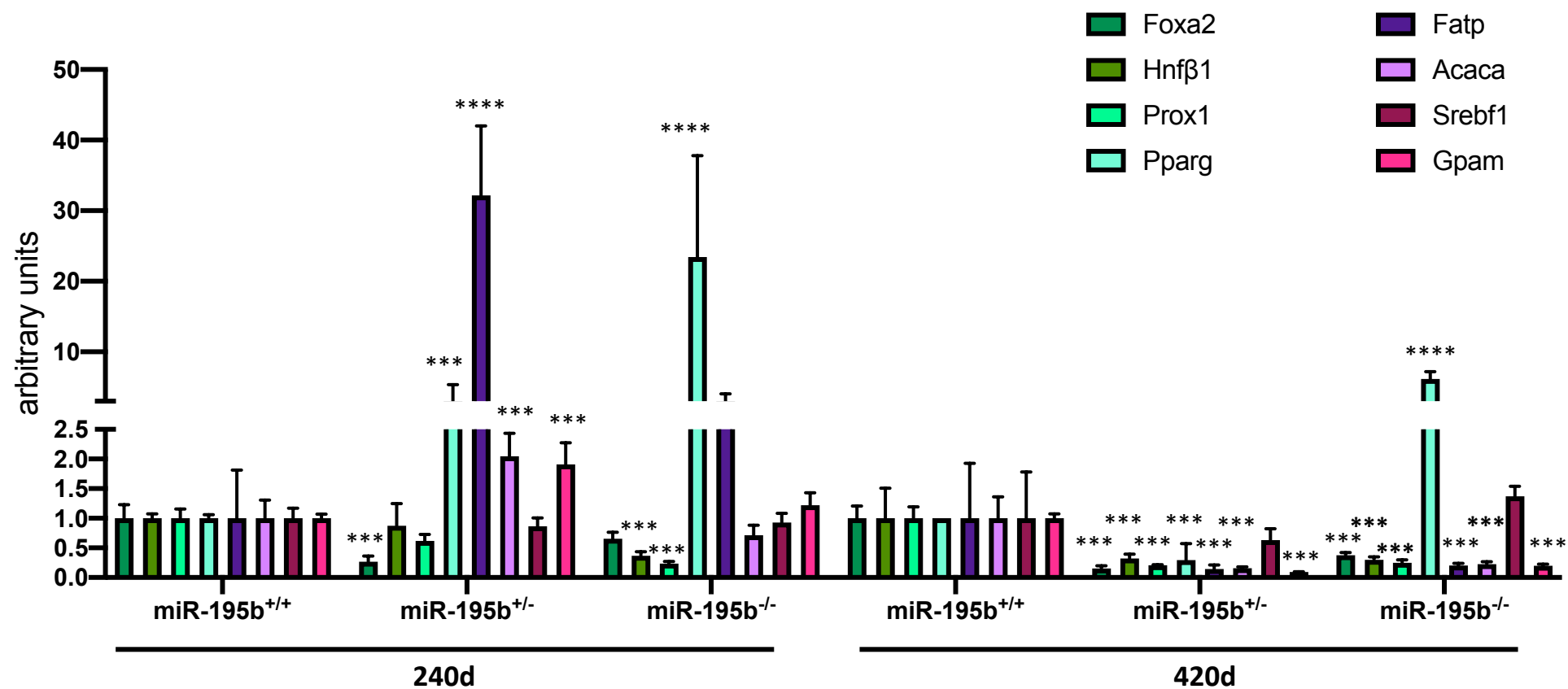

Supplement: Supplementary file 4 — Supplementary Figure S3. [file 41598_2024_51256_MOESM4_ESM.pdf]

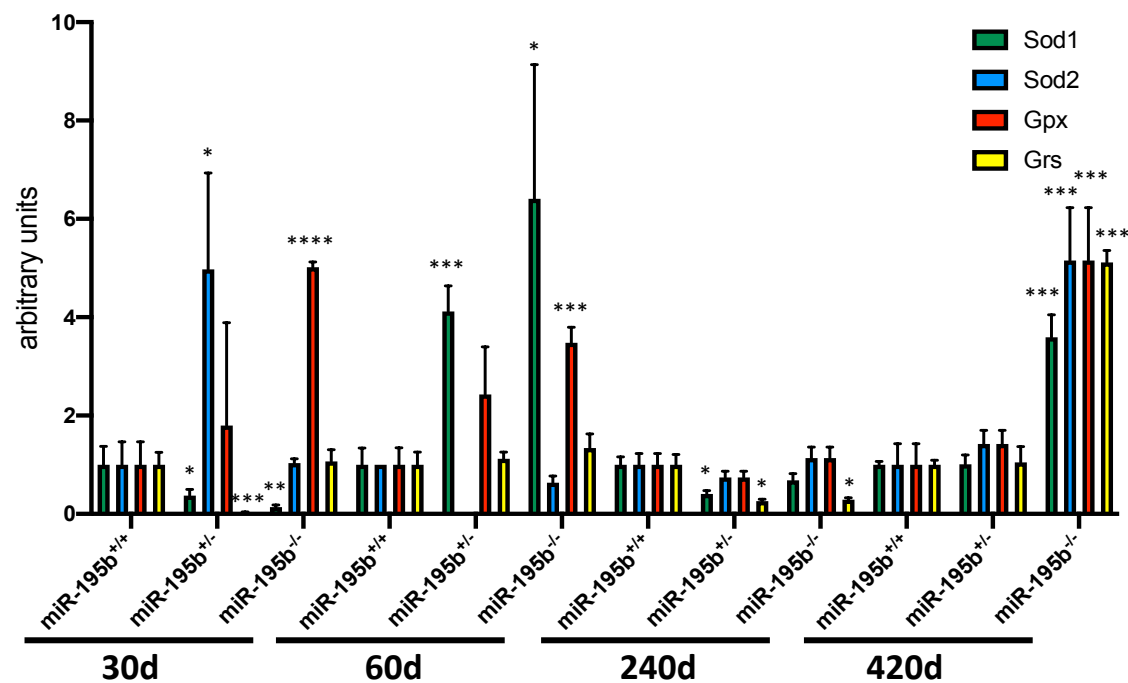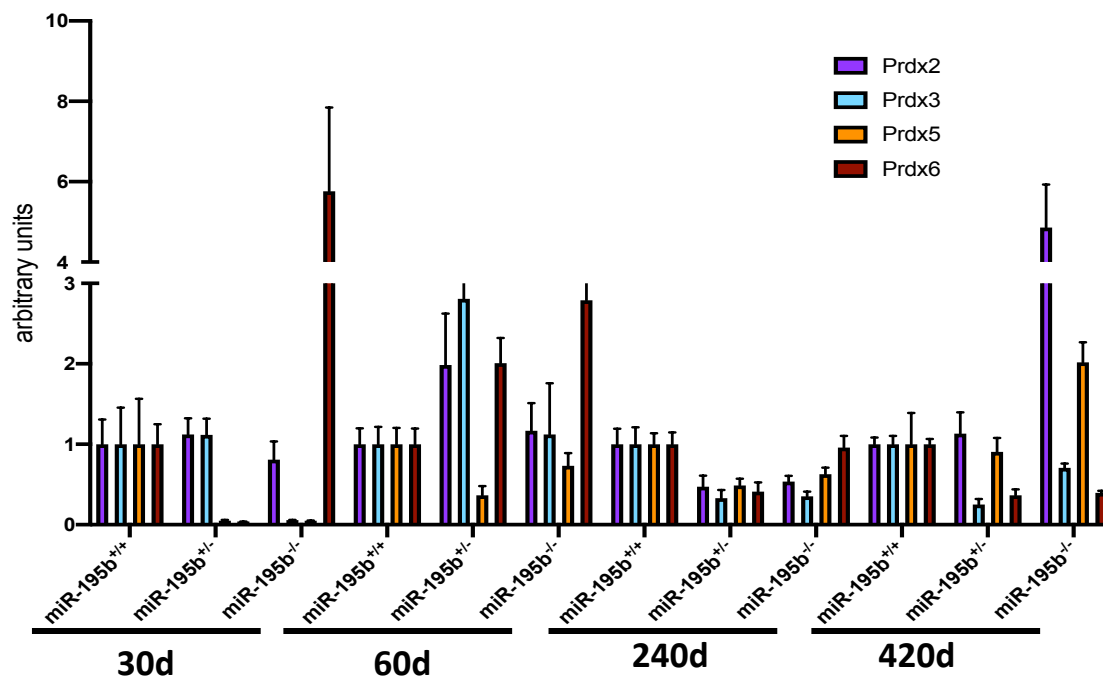

Supplement: Supplementary file 5 — Supplementary Figure S4. [file 41598_2024_51256_MOESM5_ESM.pdf]

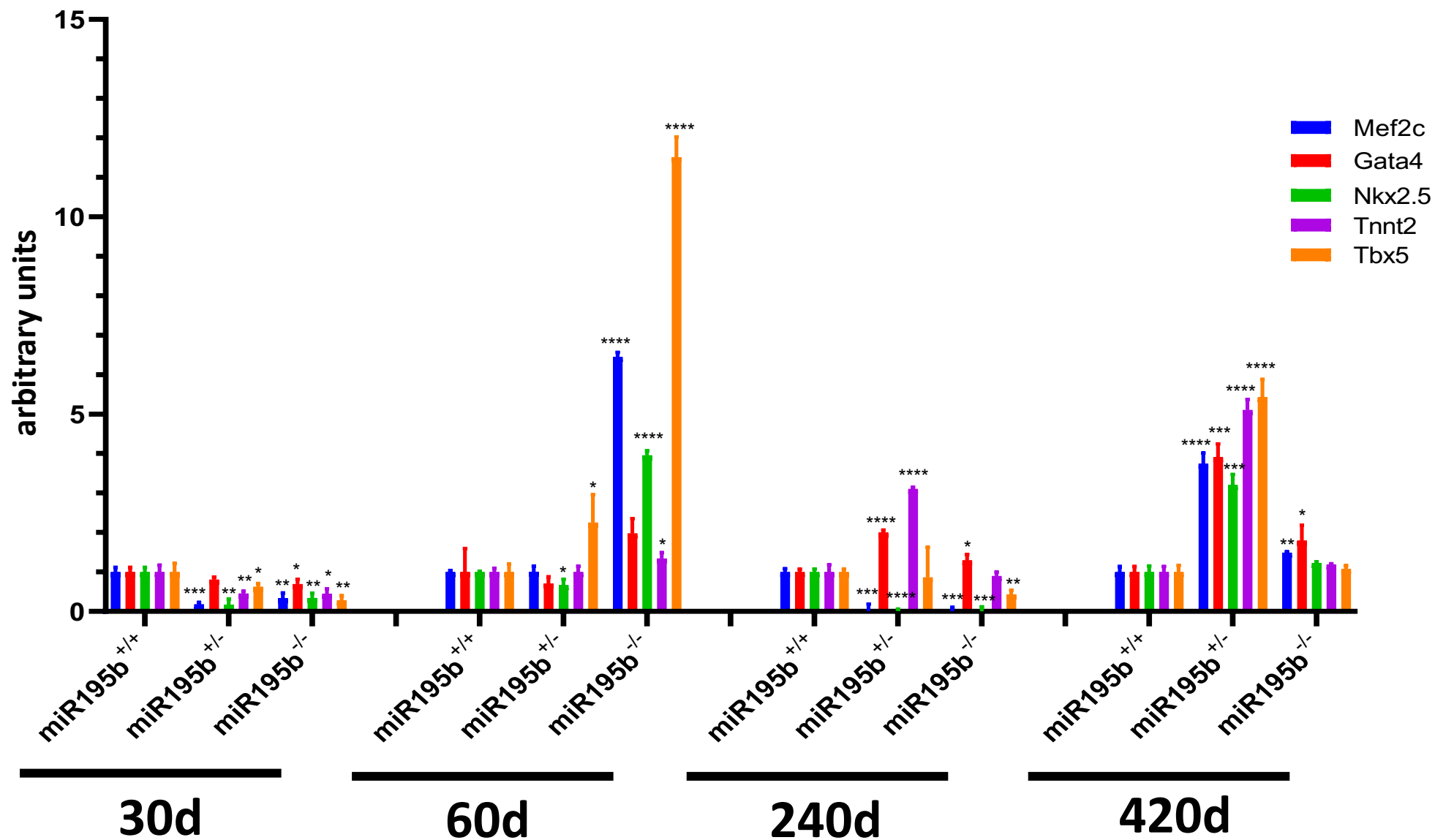

Supplement: Supplementary file 6 — Supplementary Figure S5. [file 41598_2024_51256_MOESM6_ESM.pdf]

# ROS markers

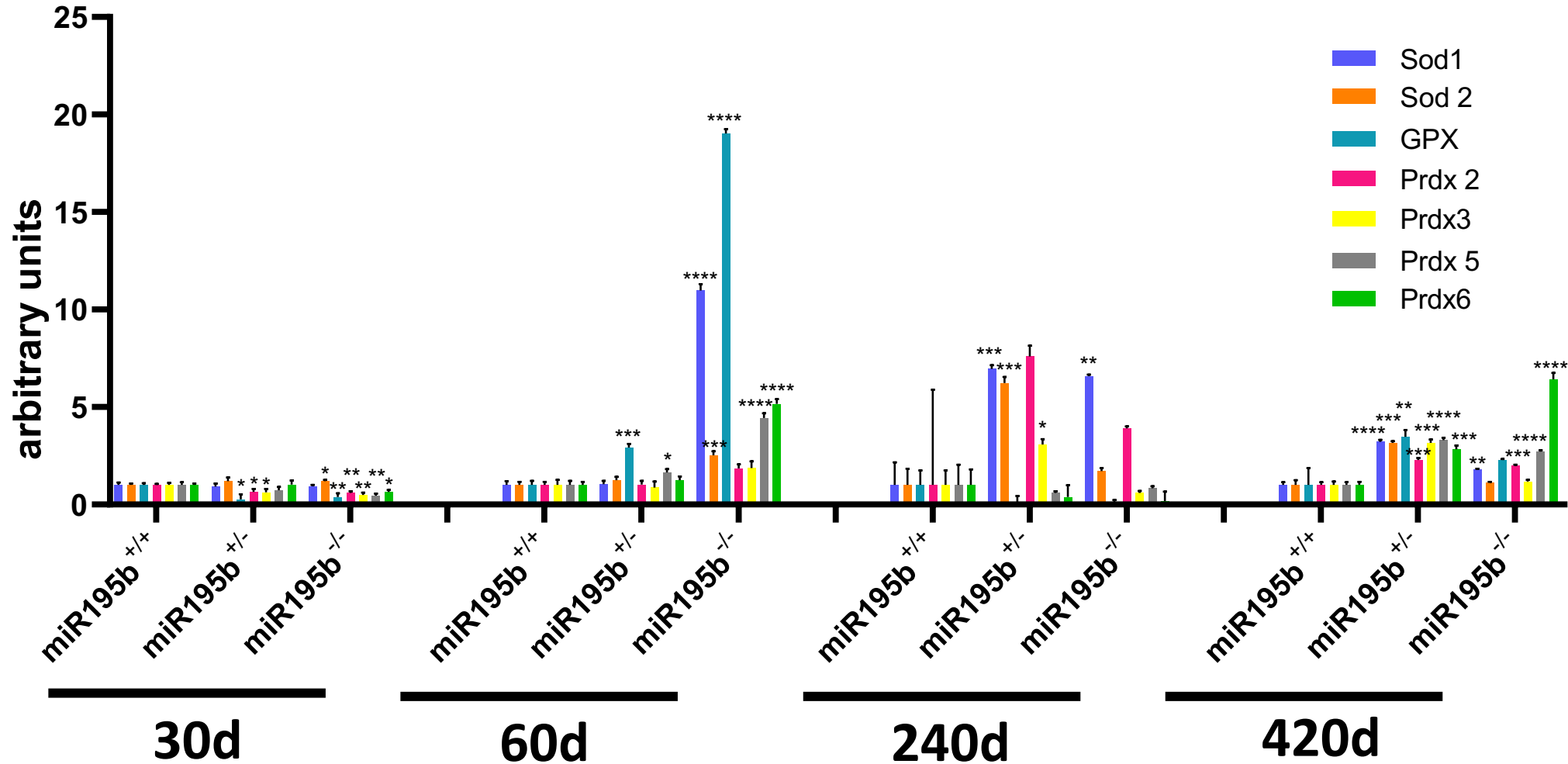

Supplement: Supplementary file 7 — Supplementary Figure S6. [file 41598_2024_51256_MOESM7_ESM.pdf]

arbitrary units

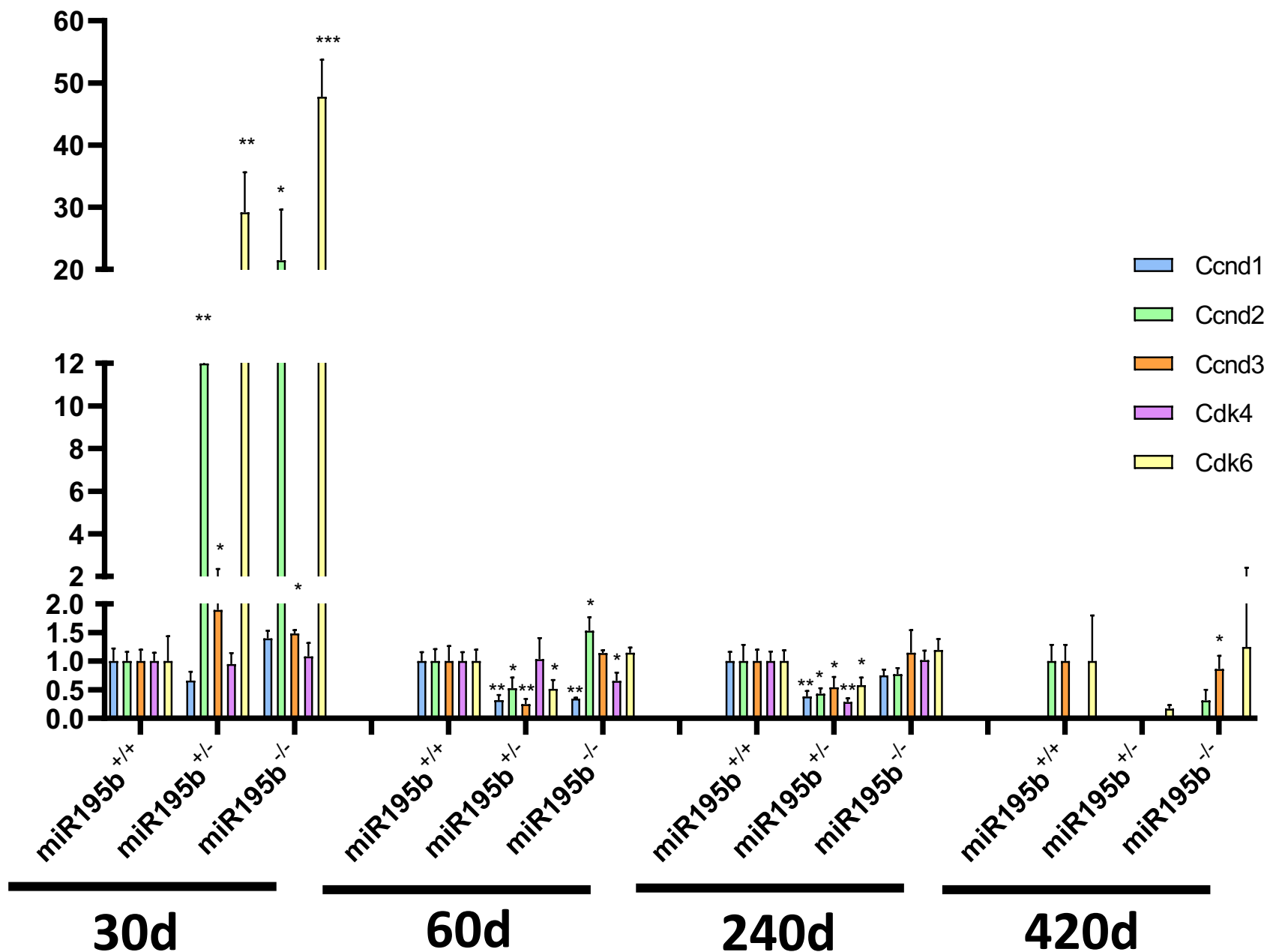

Supplement: Supplementary file 8 — Supplementary Figure S7. [file 41598_2024_51256_MOESM8_ESM.pdf]

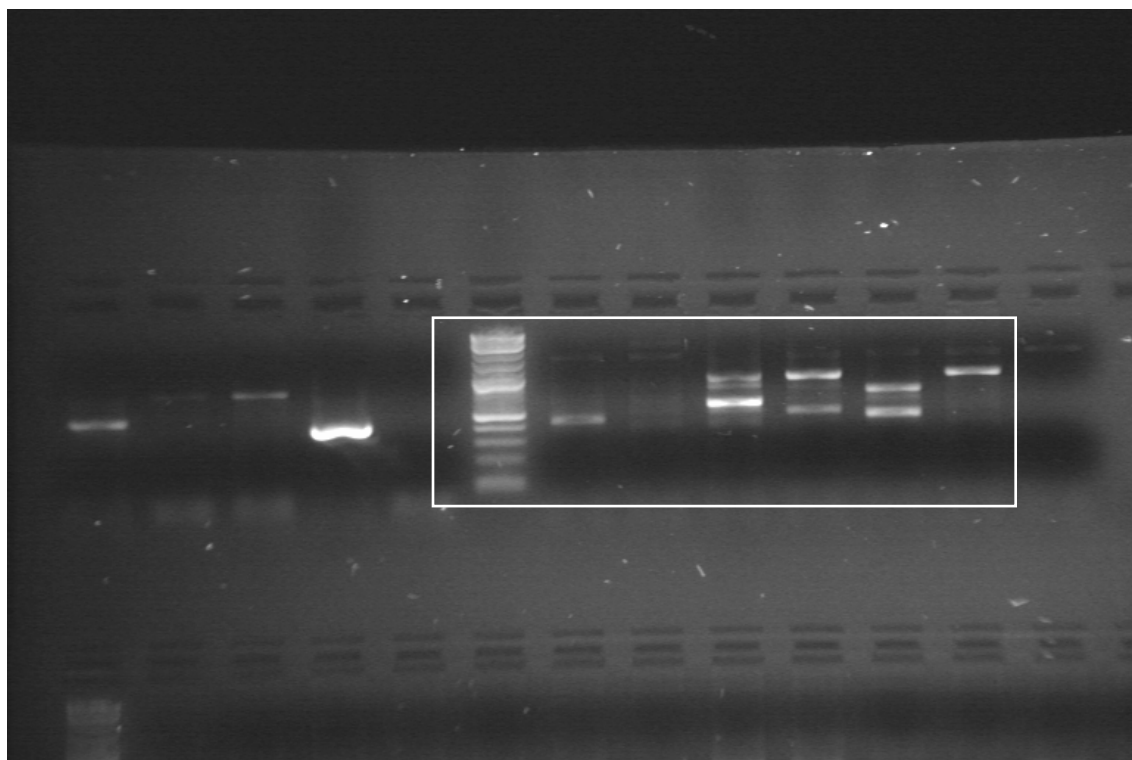

Uncropped gel (Supplementary Figure 1 B)

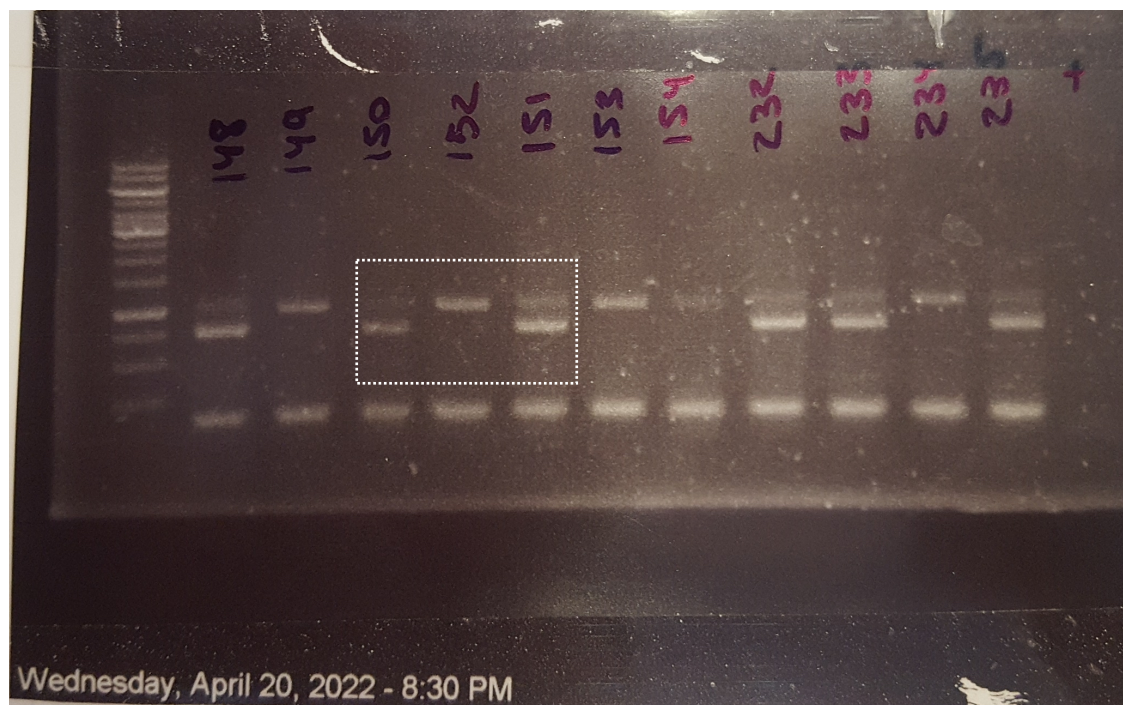

Uncropped gel (Supplementary Figure 1 C)

Supplement: Supplementary file 9 — Supplementary Figure S8. [file 41598_2024_51256_MOESM9_ESM.pdf]
