# Supplementary material for: miR-195b is required for proper cellular homeostasis in the elderly
Source: Sci Rep. 2024 Jan 8;14:810. doi: 10.1038/s41598-024-51256-8 (PMC10774362; doi:10.1038/s41598-024-51256-8)
Supplement: Supplementary file 10 — Supplementary Table S1. [file 41598_2024_51256_MOESM10_ESM.pdf]

Supplementary Table 1

mRNA primers		
Gene	Species	Sequences
Pparg	Mus musculus	GGAAGCCCTTTGGTGACTTT<br>ACGTGCTCTGTGACGATCTG
Foxa2	Mus musculus	CATCCGACTGGAGCAGCTA<br>CGGACATGCTCATGTATGTGT
Hnfb1	Mus musculus	TACAGCCCGTCCAGTTCTCT<br>TGGAAGACATGCTGGTGAGA
Prox1	Mus musculus	GCTATACCGAGCCCTCAACA<br>TGTAAATGGCCTTCTTCCAG
Fatp	Mus musculus	TGAGAACTTGCCACCGTATG<br>GGCAGGTAGGCCCCTATATC
Gapdh	Mus musculus	TCTTGCTCAGTGTCCTTGCTGG<br>TCCTGGTATGACAATGAATACGC
Srebf1	Mus musculus	CCTACATGAGGCCACAGCTC<br>CAGATAGCAGGATGCCAACA
Acaca	Mus musculus	CAGCAGGGACTATGTCCTGAA<br>TGGAAGGGGAATCCATAGTG
Gpam	Mus musculus	CGTGAAGAACGCTGTGAAAA<br>CAGCACCACAAAACTCAGAA
Sod1	Mus musculus	GAGACCTGGGCAATGTGACT<br>GTTTACTGCGCAATCCAAT
Sod2	Mus musculus	CCGAGGAGAAGTACCACGAG<br>GCTTGATAGCCTCCAGCAAC
Gpx	Mus musculus	ATCAGTTCGGACACCAGGAG<br>CATTCCGCAGGAAGGTAAAG
Gsr	Mus musculus	ACCACGAGGAAGACGAAATG<br>GGTGACCAGCTCCTCTGAAG
Prdx2	Mus musculus	CTTCGCCAGATCACAGTCAA<br>AAATCCAAGCTTCAGGCTCA
Prdx3	Mus musculus	TGGACACCAGAGTCCCCTAC<br>TCAAGGCATTGGAAGGATTC
Prdx5	Mus musculus	TGGGAAGGCGACAGACTTA<br>CAGGGCCTCAGAGTTGAGAG
Prdx6	Mus musculus	CAGGGCCTCAGAGTTGAGAG<br>GAGGGTGGGAACTACCATCA
Cnnd1	Mus musculus	CGGATGAGAACAAGCAGACC<br>AGGGTGGGTTGGAAATGAAC
Cnnd2	Mus musculus	TTCATTGAGCACATCCTTCG<br>TTCCAGTTGCAATCATCGAC
Cnnd3	Mus musculus	TAGGCGCCTGCTCTATGTCT<br>ATCTGTGGGAGTGCTGGTCT
Cdk4	Mus musculus	GATTGCCTCCAGAAGACGAC<br>TTGTGCAGGTAGGAGTGCTG
Cdk6	Mus musculus	AATCTGCTCAACCCATCGAG<br>GTTGGATGGCAGGTGAGAGT
ATF6	Mus musculus	TACCACCCACAACAAGACCA<br>TGATGATCCCGGAGATAAGG
IRE1	Mus musculus	CGAATAGAAAAGGAGGCCTTG<br>CTCGGAGGAGGTCTCTCACA
GATA4	Mus musculus	GCAGCAGCAGTGAAGAGATG<br>GCGATGTCTGAGTGACAGGA

Tbx5	Mus musculus	GAGCACAGCCAAATTTACCA<br>CCGAGCGATAGAAGGTGTCT
Nkx 2.5	Mus musculus	TTGGCGTCGGGGACTTGAAC<br>GGTGGGTGTGAAATCCGAGGGAC
Mef2c	Mus musculus	CTGGCAACAGCAACACCTAC<br>GAAGGCAGGGAGAGATTTGA

Luciferase Assay primers		
Gene	Species	Sequences
Stim1_3'UTR	Mus musculus	GGGACTAGTCTGCTCATCCTTGGTCCTTC<br>GGAAGCTTGGGGAGTAATGGGAGAGAGG
ATF6_3'UTR	Mus musculus	GGGACTAGTCCACCTGTGGACTCCATATTC<br>GGACTAGTGACCCTGCCAGACTGAA
Cyclin D1_3'UTR	Mus Musculus	GGGACTAGTGCCATCCAAACTGAGGAAAA<br>GGAAGCTTGATCCTGGGAGTCATCGGTA
Cyclin D2_3'UTR	Mus Musculus	GGGACTAGTGAGAGTGTGCGTACCTGCAA<br>GGAAGCTTGAGTATGCGACGGAGAGACC
Cyclin D3_3'UTR	Mus Musculus	GGGACTAGTTCCGTACTCCAGCTGCTCTT<br>GGAAGCTTCCAACCTAACCCTGCTCTGA

microRNAs primers		
Gene	Species	Sequences
Hsa-miR 15a	Human/Mouse	UAGCAGCACAUAAUGGUUUGUG
Has-miR-15b	Human/Mouse	UAGCAGCACAUCAUGGUUUACA
Hsa-miR 16-5p	Human/Mouse	UAGCAGCACGUAAAUAUUGGCG
Hsa-miR 195-3p	Human/Mouse	CCAAUAUUGGCUGUGCUGCUCC
Hsa-miR 195-5p	Human/Mouse	UAGCAGCACAGAAAUAUUGGC
Hsa-miR 195b	Human/Mouse	UAGCAGCACAGAAAUAGUAGAA
